# Contribution of Syndecans to the Cellular Entry of SARS-CoV-2

**DOI:** 10.3390/ijms22105336

**Published:** 2021-05-19

**Authors:** Anett Hudák, Annamária Letoha, László Szilák, Tamás Letoha

**Affiliations:** 1 Pharmacoidea Ltd., H-6726 Szeged, Hungary; anett.hudak@pharmacoidea.eu (A.H.); laszlo.szilak@gmail.com (L.S.); 2 Department of Medicine, Albert Szent-Györgyi Clinical Center, Faculty of Medicine, University of Szeged, H-6725 Szeged, Hungary; letohadr@gmail.com; 3 Szilak Laboratories, Bioinformatics and Molecule-Design, H-6723 Szeged, Hungary

**Keywords:** coronaviruses, SARS-CoV-2, spike protein, cellular entry, syndecans

## Abstract

The severe acute respiratory syndrome coronavirus 2 (SARS-CoV-2) is a novel emerging pathogen causing an unprecedented pandemic in 21st century medicine. Due to the significant health and economic burden of the current SARS-CoV-2 outbreak, there is a huge unmet medical need for novel interventions effectively blocking SARS-CoV-2 infection. Unknown details of SARS-CoV-2 cellular biology hamper the development of potent and highly specific SARS-CoV-2 therapeutics. Angiotensin-converting enzyme-2 (ACE2) has been reported to be the primary receptor for SARS-CoV-2 cellular entry. However, emerging scientific evidence suggests the involvement of additional membrane proteins, such as heparan sulfate proteoglycans, in SARS-CoV-2 internalization. Here, we report that syndecans, the evolutionarily conserved family of transmembrane proteoglycans, facilitate the cellular entry of SARS-CoV-2. Among syndecans, the lung abundant syndecan-4 was the most efficient in mediating SARS-CoV-2 uptake. The S1 subunit of the SARS-CoV-2 spike protein plays a dominant role in the virus’s interactions with syndecans. Besides the polyanionic heparan sulfate chains, other parts of the syndecan ectodomain, such as the cell-binding domain, also contribute to the interaction with SARS-CoV-2. During virus internalization, syndecans colocalize with ACE2, suggesting a jointly shared internalization pathway. Both ACE2 and syndecan inhibitors exhibited significant efficacy in reducing the cellular entry of SARS-CoV-2, thus supporting the complex nature of internalization. Data obtained on syndecan specific in vitro assays present syndecans as novel cellular targets of SARS-CoV-2 and offer molecularly precise yet simple strategies to overcome the complex nature of SARS-CoV-2 infection.

## 1. Introduction

The severe acute respiratory syndrome coronavirus 2 (SARS-CoV-2) is a beta-coronavirus initially emerging in China and then rapidly spreading throughout the world, becoming a significant threat to human health [1,2,3,4,5,6,7,8]. On 13 March, the World Health Organization (WHO) declared Europe the epicenter of the SARS-CoV-2 pandemic [9]. Caused by SARS-CoV-2 infection, the coronavirus disease 2019 (COVID-19) poses specific challenges for adequate and effective treatment to avoid the onset of severe clinical manifestations [10]. Currently, there is no specific antiviral therapy against SARS-CoV-2 infection [11]. Several investigational agents have been described in observational studies or used based on in vitro or extrapolated evidence [12,13]. Among the applied anti-COVID-19 therapeutics, remdesivir, an antiviral agent originally developed against Ebola infection, shows one of the most promising clinical efficacy in attenuating the severity of COVID-19 [14,15]. Given the high mortality despite remdesivir, novel and more efficient combinatory strategies should be developed to improve patient outcomes in COVID-19 [15].

Coronaviruses are endowed with a high tendency to spread from animals to humans, enabling cross-species transmission and facilitating severe outbreaks [5,16,17,18,19]. The capacity of coronaviruses for cross-species transmission also supports the need to develop highly efficient yet safe therapeutics and prophylactics to tackle current and future pandemics [6,20,21,22]. Development of efficient therapeutics against COVID-19 is hampered by the unknown details of SARS-CoV-2 cellular biology, which is greatly postulated on previous studies with SARS-CoV, a coronavirus strain responsible for the first SARS outbreak in 2002–2003 [10]. Exploring the specific molecular events driving SARS-CoV-2 infection is critical for developing novel and specific medicines against COVID-19. SARS-CoV-2 is transmitted by multiple means, including liquid droplets, aerosol particles and fomites [23,24]. Once SARS-CoV-2 enters the nasal cavity, it binds to epithelial cells and migrates down the respiratory tract while triggering a robust immune response [10,25]. The angiotensin-converting enzyme 2 (ACE2) has been identified as the primary entry receptor for both SARS-CoV-2 and SARS-CoV [10,26,27,28]. However, scientific evidence shows that endocytosis of SARS-CoV also occurs through a novel, clathrin- and caveolae-independent endocytic pathway, mediated by attachment to cell surface heparan sulfate proteoglycans (HSPGs) [29,30,31,32]. Meanwhile, it has also been revealed that the S1 subunit of the SARS-CoV-2 spike protein, the subunit responsible for cellular attachment, contains the heparin-binding core motif PRRAR [33,34,35,36] (Supplementary Figure S1). According to the most recent SARS-CoV-2 infection models, viral attachment and infection involve the formation of a complex between heparan sulfate (HS) and ACE2 [37].

HSPGs are glycoproteins containing one or more covalently attached HS chains, a type of glycosaminoglycan (GAG) [38,39]. The evolutionarily highly conserved syndecans (SDCs) are the only transmembrane HSPG family and possess essential roles in cell interactions, adhesion, migration and signaling [40,41,42]. SDCs share a similar structure: a one-span and highly conserved transmembrane domain (TM) and a relatively short cytoplasmic domain (CD) [43,44]. The extracellular domain (ectodomain) of SDCs is more diverse, containing HS attachment sites with GAG side chains [45,46]. Through their highly sulfated GAG chains, SDCs interact with a myriad of extracellular ligands, transmitting extracellular signals intracellularly [40,45,46,47,48]. Besides cell signaling, SDCs also mediate their ligands’ intracellular transport [45,46,47]. During SDC-mediated endocytosis, ligand-mediated clustering of SDCs induces the redistribution of SDCs to lipid rafts and stimulation of a lipid raft-dependent clathrin- and caveolae-independent internalization of the SDC-ligand complex [46,47,49]. As ligands internalized through SDC-mediated endocytosis can avoid lysosomal degradation, several parasites, including viruses and bacteria, utilize SDCs as shuttles to enter the cells [50,51,52,53,54,55,56].

Members of the SDC family show tissue-specific expression: SDC1 is expressed on epithelial and plasma cells, SDC2 on endothelial cells, SDC3 in the neurons, while SDC4 is ubiquitous [43,44,53,57]. The BioGPS gene expression database (http://biogps.org, accessed on 10 March 2020) indicates a high expression of SDC4 in human lung cells [53,58]. In the lung, SDCs contribute to the balanced inflammation progression [59]. During SARS-CoV and SARS-CoV-2 infection, certain chemokines such as the C-X-C motif chemokine ligand 10 (CXCL10) might be predictive of the subsequent clinical course [60]. CXCL10 and SDC4 display a close interaction during lung inflammation, while SDC1 is essential to limit inflammation and lung injury after influenza infection [61,62,63]. Viruses targeting SDCs in the lung could thus interfere with SDC-dependent signaling, influencing the infection’s inflammatory response. The involvement of SDC4 in antiviral signaling regulation has also been reported [56]. In their excellent paper, Lin et al. meticulously demonstrated the SDC4 inducing effect of a viral infection, along with SDC4’s influence on attenuating antiviral immunity [56].

Our research group has been focusing on exploring SDCs’ drug delivery and therapeutic potential. Our related studies with non-viral drug delivery agents, including cell-penetrating peptides (CPPs) and cationic liposomes, contributed to understanding SDCs’ capacity to deliver bioactive macromolecules into the cells [43,44,64]. Moreover, we also revealed that SDCs contribute to the seeding and prion-like spreading of pathological protein aggregates, the main molecular culprits responsible for the onset of neurodegenerative disorders [45,46]. We developed several SDC specific assays and constructs during these endeavors, enabling thorough analyses of SDCs’ interactions with potential ligands. Considering the scientific evidence on the potential involvement of SDCs in SARS-CoV-2 infection, we progressed to explore the interaction of both SARS-CoV-2 and its spike protein S1 subunit (spikeS1) with SDC isoforms. Our SDC specific transfectants enabled the quantitative analysis of SDCs’ contribution to the cellular entry of SARS-CoV-2, while structural SDC mutants allowed us to examine the interaction of the virus and its spikeS1 with various parts of the SDC ectodomain. Besides SDC specific transfectants, our studies also included the lung-specific A549 cell line that, due to its relative resistance against ACE2-mediated SARS-CoV infection, poses a challenge to the current ACE2 paradigm [65]. Utilizing SARS-CoV-2 pseudovirus (PSV) [66,67,68] encoding red fluorescent protein (RFP) as a reporter gene enabled us to explore the effect of SDCs on SARS-CoV-2-mediated gene transduction.

Overall, our findings present SDCs as essential contributors to SARS-CoV-2 cellular entry and highlight the interplay of SDCs with ACE2 during the virus’s cellular uptake. Exploring the complex molecular interplay driving SARS-CoV-2 cellular entry also helped us identify novel inhibitors of virus internalization.

## 2. Results

### 2.1. SDCs Facilitate Cellular Uptake of the SARS-CoV-2

**Figure 1:**
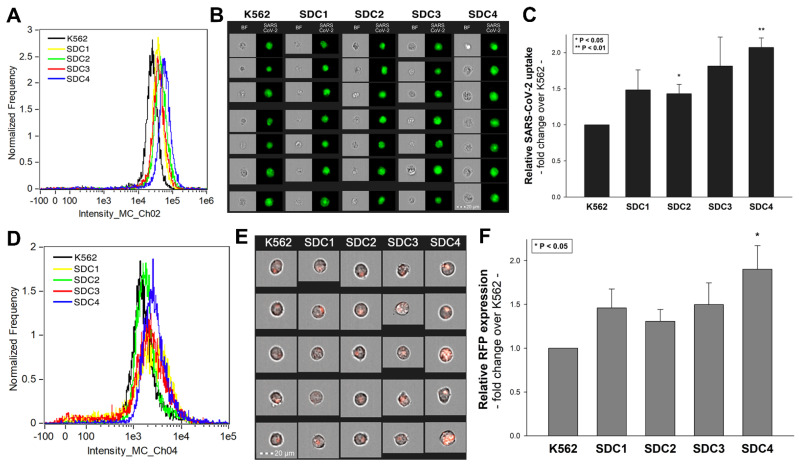
Cellular entry of SARS-CoV-2 into SDC transfectants. WT K562 cells and SDC transfectants were incubated with heat-inactivated SARS-CoV-2 (at 1 MOI) for 18 h at 37 °C. After incubation, the cells were washed, trypsinized, fixed, permeabilized and treated with antibodies specific for the spike glycoprotein (along with secondary AF 488-labeled antibodies). Cellular uptake of SARS-CoV-2 was then analyzed with imaging flow cytometry and confocal microscopy. (**A**) Representative flow cytometry histograms showing the intracellular fluorescence of SARS-CoV-2-treated WT K562 cells and SDC transfectants. (**B**) Brightfield (BF) and fluorescent cellular images of SARS-CoV-2-treated WT K562 cells and SDC transfectants. Scale bar = 20 μm. (**C**) Detected fluorescence intensities were normalized to SARS-CoV-2-treated WT K562 cells as standards. The bars represent the mean ± SEM of four independent experiments. Statistical significance vs. standards was assessed with analysis of variance (ANOVA). * *p* < 0.05; ** *p* < 0.01. (**D**–**F**) Contribution of SDCs to SARS-CoV-2 PSV-mediated gene transduction. WT K562 cells and stable SDC transfectants were incubated with 1 × 10^5^ transducing units of SARS-CoV-2 PSV-RFP. RFP expression was analyzed 72 h later with imaging flow cytometry. (**D**) Representative flow cytometry histograms showing RFP fluorescence of WT K562 cells and SDC transfectants, following 72 h incubation with SARS-CoV-2 PSV. (**E**) Cellular images of SARS-CoV-2 PSV-treated WT K562 cells and SDC transfectants as detected with imaging flow cytometry. Scale bar = 20 μm. (**F**) Detected cellular RFP intensities were normalized to SARS-CoV-2 PSV-treated WT K562 cells as standards. The bars represent the mean ± SEM of four independent experiments. Statistical significance vs. standards was assessed with ANOVA. * *p* < 0.05.

SDC isoforms were created in K562 cells, a human myeloid leukemia cell line lacking endogenous HSPGs except for minor amounts of endogenous betaglycan [55,69]. K562 cells also express no detectable levels of caveolin-1, the main component of caveolae [70]. Due to their limited HSPG expression and inability to form caveolae, the source of caveolar endocytosis, K562 cells offer ideal cellular models to study the contribution of SDCs to cellular uptake of ligands without the interfering effects of other HSPGs or caveolae-mediated endocytosis [45,46]. As HS has already been established as a primary binding site for several viruses [52], including SARS-CoV [32], stable SDC transfectants created in K562 cells were standardized according to their HS content (Supplementary Figure S2) [45,46] (it is worth noting that SDC transfection did not induce statistically significant changes in ACE2 expression (Supplementary Figure S3)). Thus, SDC transfectants with an equal amount of HS expression were selected and, along with WT K562 cells, treated with heat-inactivated SARS-CoV-2 (at 1 MOI (multiplicity of infection)). After 18 h of incubation, the virus’s cellular uptake was detected by incubating the SARS-CoV-2-treated, fixed and permeabilized cells with Alexa Fluor 488 (AF 488) labeled antibodies specific for SARS-CoV-2’s spike glycoprotein. For imaging flow cytometry analyses, surface-attached SARS-CoV-2 was removed with trypsinization, enabling the internalized viral particles’ measurement only [71,72]. Imaging flow cytometry analyses revealed increased uptake of SARS-CoV-2 into SDC transfectants (Figure 1A–C). Among SDCs, SDC4 increased SARS-CoV-2 uptake the most (*p* < 0.01) (incubating the cells with the AF 488-labeled secondary antibodies did not result in any statistically significant difference in cellular fluorescence of applied WT K562 cells and SDC transfectants, showing that no unspecific binding influenced the detected difference in fluorescence intensities of SARS-CoV-2-treated cells (Supplementary Figure S4). Treating WT K562 cells and SDC transfectants with the SARS-CoV-2 pseudovirus (SARS-CoV-2 PSV), a recombinant pseudotyped lentiviral particle carrying the SARS-CoV-2 spike protein and encoding the red fluorescent protein (RFP) as a reporter gene [29,66,67,68], delivered similar results as uptake studies with the heat-inactivated SARS-CoV-2. Namely, SDC transfectants all increased SARS-CoV-2 PSV-mediated RFP transduction (Figure 1D–F). Compared to PSV-treated WT K562 cells, the increase in RFP transduction was significant (*p* < 0.05) only in the case of SDC4 transfectants (Figure 1F). PSV studies thus showed that besides facilitating SARS-CoV-2 uptake, SDC4-mediated cellular entry also maintains the biological activity (i.e., the gene transduction ability) of the virus.

**Figure 2:**
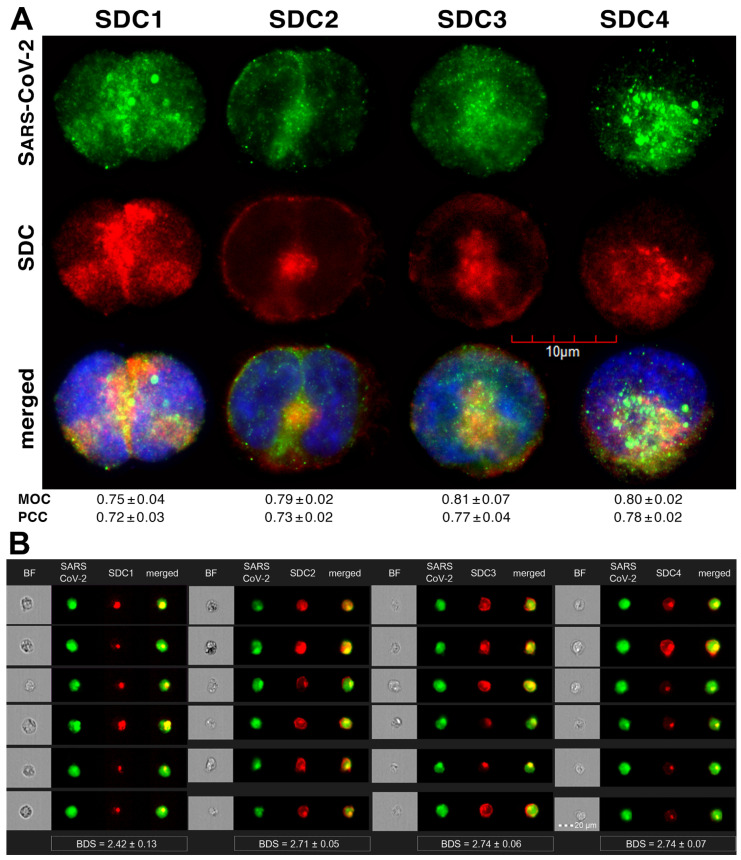
SARS-CoV-2 colocalizes with SDCs during cellular entry. WT K562 cells and SDC transfectants were incubated with heat-inactivated SARS-CoV-2 (at 1 MOI) for 18 h at 37 °C. After incubation, the cells were washed, trypsinized, fixed, permeabilized and treated with antibodies specific for the spike glycoprotein (along with secondary AF 488-labeled antibodies) and APC-labeled SDC antibodies. Colocalization of SARS-CoV-2 with SDCs was analyzed with confocal microscopy and imaging flow cytometry. (**A**) Microscopic analyses of SARS-CoV-2 and SDC colocalization. Representative images of three independent experiments are shown. Scale bar = 10 μm. The MOC and PCC ± SEM for the overlap and colocalization of SDC with SARS-CoV-2 (indicated below the images) were calculated by analyzing 15 images with an average of 10 cells in each image (from 3 separate samples). (**B**) BF and fluorescent images of SARS-CoV-2-treated SDC transfectants. Scale bar = 20 μm. The indicated BDS values of SARS-CoV-2 and SDCs represent mean ± SEM of four independent experiments.

Colocalization studies revealed significant colocalization between SARS-CoV-2 and SDCs, suggesting the same route SDCs and SARS-CoV-2 follow during cellular entry (Figure 2A,B and Supplementary Figure S5). The Mander’s overlap and Pearson correlation coefficients (MOC and PCC, respectively) for SDCs and SARS-CoV-2 exceeded 0.7, indicating significant colocalization (Figure 2A and Supplementary Figure S5). The colocalization of SARS-CoV-2 with SDCs during virus entry was also confirmed with imaging flow cytometry (Figure 2B). The Bright Detail Similarity (BDS) score of colocalization between the fluorescent signals of the SDCs and SARS-CoV-2 also showed a high degree of colocalization (generally, a BDS score of two or greater represents a high degree of overlap [73]), especially in SDC3 and 4 transfectants (Figure 2B).

### 2.2. Contribution of Various Parts of the SDC4 Ectodomain to SARS-CoV-2 Uptake

**Figure 3:**
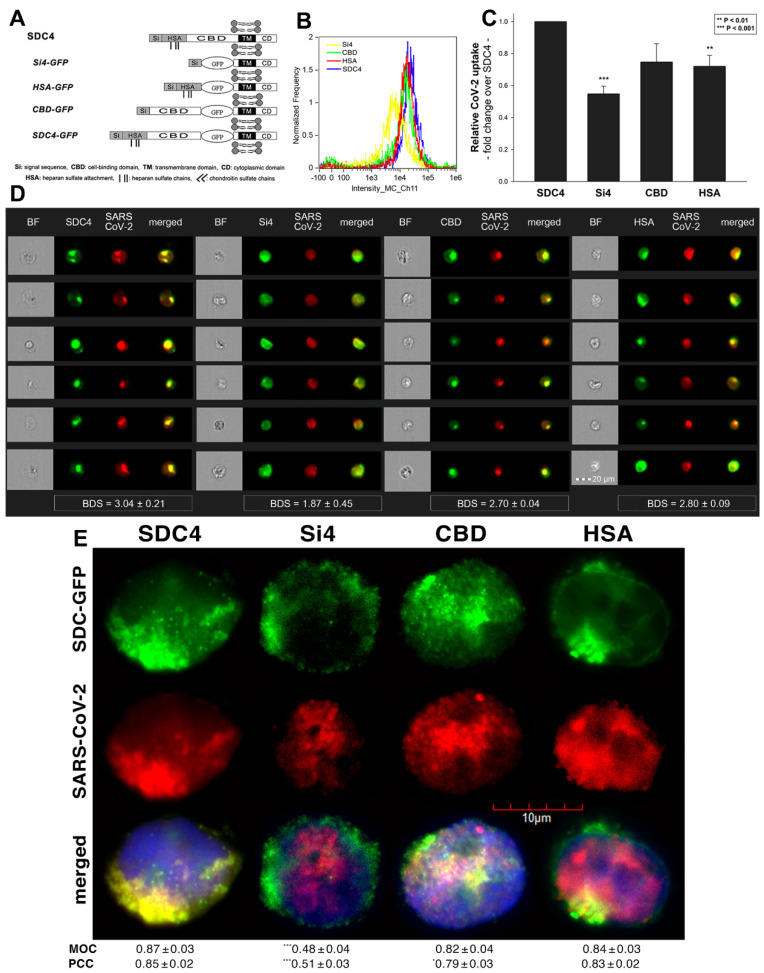
Contribution of the various parts of the SDC4 ectodomain to SARS-CoV-2 uptake. GFP-tagged SDC4 mutants incubated with SARS-CoV-2 (at 1 MOI) for 18 h were fixed, permeabilized and treated with specific and AF 633-labeled SARS-CoV-2 antibodies. Cellular uptake was analyzed with imaging flow cytometry and confocal microscopy. (**A**) Schematic representation of the applied SDC4 mutants. (**B**) Representative flow cytometry histograms showing the intracellular fluorescence of SARS-CoV-2-treated SDC4 transfectants and mutants. (**C**) Detected fluorescence intensities were normalized to SARS-CoV-2-treated transfectants expressing WT SDC4 as standards. The bars represent the mean ± SEM of four independent experiments. Statistical significance vs. standards was assessed with ANOVA. ** *p* < 0.01; *** *p* < 0.01. (**D**) BF and fluorescent images of SARS-CoV-2-treated SDC4 mutants. Scale bar = 20 μm. The indicated BDS values of SARS-CoV-2 and SDCs represent the mean ± SEM of four independent experiments. Statistical significance between the SDC4 mutants was assessed with ANOVA. (**E**) Confocal microscopic visualization of SARS-CoV-2-treated SDC4, Si4, CBD and HSA transfectants. Scale bar = 10 μm. MOC ± SEM and PCC ± SEM for the overlap and colocalization of SARS-CoV-2 with SDC4, Si4, CBD and HSA (indicated below the images) was calculated by analysis of 15 images with ~10 cells in each image (from 3 separate samples). Statistical significance vs. SARS-CoV-2-treated transfectants expressing WT SDC4 (standards) was assessed with ANOVA. * *p* < 0.05, *** *p* < 0.001.

Studies on isoform-specific SDC cell lines demonstrated that SDCs increase cellular uptake of SARS-CoV-2. Among SDCs, SDC4 facilitated cellular uptake of SARS-CoV-2 the most. To investigate the molecular mechanisms driving SARS-CoV-2’s interaction with the SDC4 ectodomain, heat-inactivated SARS-CoV-2 was incubated (at 1 MOI) with transfectants expressing various SDC4 structural mutants (Figure 3A). Deletion mutant Si4 possesses a truncated SDC4 extracellular domain made of only the short signal sequence (Si), while mutant CBD has a mutated ectodomain containing only the cell-binding domain (CBD) and Si, but no HS attachment (HSA) site or HS chains [43,44,45,46]. We also applied the deletion mutant HSA with an ectodomain comprising the HSA site and HS chains (and the Si) but no CBD [43,44,45,46]. To readily detect their expression, all of the SDC4 mutants—along with WT SDC4—were tagged with GFP and expressed in K562 cells [43,44,45,46] (as shown in Supplementary Figure S6, expression of the SDC4 mutants did not influence ACE2 expression). Clones with an equal extent of SDC expression were selected and treated with SARS-CoV-2. After incubation, the cells were trypsinized to remove extracellularly attached viral particles [71,72]. The cells were then fixed, permeabilized and treated with fluorescently (AF 633) labeled antibodies specific for the spike glycoprotein of SARS-CoV-2. Fluorescence was then analyzed with imaging flow cytometry and confocal microscopy. Imaging flow cytometry revealed that both the HSA and CBD of SDC4 has a significant role in interacting with SARS-CoV-2 (Figure 3B–D). Namely, deleting both the CBD and the HSA with HS chains reduced cellular uptake of SARS-CoV-2, as shown by the markedly reduced intracellular fluorescence detected in Si4 mutants (Figure 3B–D). However, the insignificant reduction in the cellular fluorescence of SARS-CoV-2-treated CBD mutants showed that the CBD plays an important role in interacting with the virus. Microscopic colocalization also showed substantial colocalization of SARS-CoV-2 with either the HSA or CBD mutants (Figure 3E and Supplementary Figure S7), both with MOC and PCC values around 0.8, demonstrating that SARS-CoV-2 could attach to both the HS chains or the CBD of SDC4. Contrary to CBD and HSA mutants, the MOC values measured on Si4 mutants showed significant (i.e., *p* < 0.001) reduction vs. WT SDC4, thus highlighting the importance of CBD and HSA in the interactions with SARS-CoV-2. Overall, our studies with SDC4 deletion mutants revealed that besides the polyanionic HS chains, SARS-CoV-2 also interacts with the CBD of SDC4, highlighting the importance of the HS-independent parts of the SDC4 core protein (incubating the cells with the AF 633-labeled secondary antibodies did not induce any difference in fluorescence among the applied SDC4 transfectants and SDC4 mutants, showing that no unspecific binding influenced the difference in the detected fluorescence intensities in SARS-CoV-2-treated cells (Supplementary Figure S8)).

### 2.3. Cellular Internalization of SARS-CoV-2 into A549 Cells

**Figure 4:**
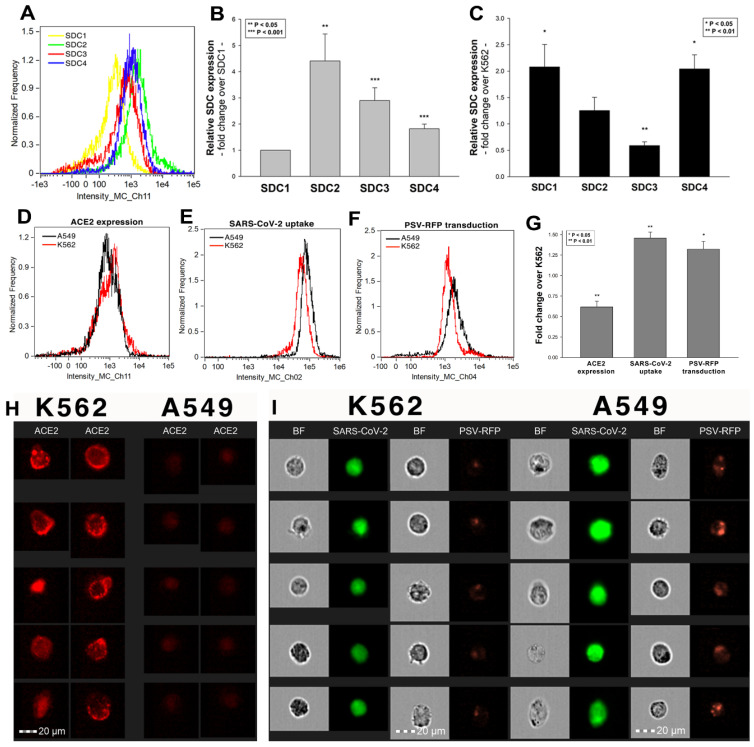
The difference of SARS-CoV-2 internalization in A549 and K562 cells. WT A549 and K562 cells were incubated with heat-inactivated SARS-CoV-2 (at 1 MOI) for 18 h at 37 °C. After incubation, the cells were washed, trypsinized, fixed, permeabilized and treated with antibodies specific for the spike glycoprotein (along with AF 488-labeled secondary antibodies). Cellular uptake of SARS-CoV-2 was then analyzed with imaging flow cytometry. Other A549 and K562 cells were also incubated with 1 × 10^5^ transducing units of SARS-CoV-2 PSV-RFP for 72 h. RFP expression was then measured with imaging flow cytometry. To investigate the effect of ACE2 and SDC expression on SARS-CoV-2 uptake, ACE2 and SDC expression of WT A549 and K562 cells (all untreated with SARS-CoV-2) were also analyzed with flow cytometry by using fluorescently labeled antibodies specific for ACE2 and SDC isoforms. (**A**) Representative flow cytometry histograms showing the expression levels of SDC isoforms in WT A549 cells. (**B**) Detected SDC expression levels in A549 cells were normalized to that of SDC1. The bars represent the mean ± SEM of nine independent experiments. Statistical significance vs. SDC1 expression was assessed with ANOVA. ** *p* < 0.01; *** *p* < 0.001. (**C**) SDC expression of WT A549 and K562 cells was measured with flow cytometry. Detected SDC expression levels in A549 cells were normalized to that of K562 cells as standards. The bars represent the mean ± SEM of three independent experiments. Statistical significance vs. standards was assessed with ANOVA. * *p* < 0.05; ** *p* < 0.01. (**D**) Representative flow cytometry histograms showing the ACE2 expression levels in WT K562 and A549 cells. (**E**) Representative flow cytometry histograms showing the intracellular fluorescence of SARS-CoV-2-treated WT K562 and A549 cells. (**F**) Representative flow cytometry histograms showing the RFP fluorescence of SARS-CoV-2 PSV-treated WT K562 and A549 cells. (**G**) ACE2 and RFP expression and SARS-CoV-2 internalization levels were normalized to those of WT K562 cells as standards. The bars represent the mean ± SEM of three independent experiments. Statistical significance vs. standards (WT K562 cells) was assessed with ANOVA. * *p* < 0.05; ** *p* < 0.01. (**H**) Fluorescent images representing the ACE2 expression of K562 and A549 cells. Scale bar = 20 μm. (**I**) BF and fluorescent images of SARS-CoV-2- and SARS-CoV-2 PSV-treated K562 and A549 cells. Scale bar = 20 μm.

After assessing the interaction of SARS-CoV-2 with the SDC4 ectodomain, we conducted studies on A549 cells, a human airway epithelia with a reportedly low level of endogenous ACE2 expression [65]. As overexpression of ACE2 did not render to A549 cells to support SARS-CoV replication, A549 cells offer an ideal cellular model to study novel pathways for coronavirus entry [74]. Exploring the SDC expression profile of A549 cells showed modest yet detectable levels of SDCs (Figure 4A,B). In terms of ACE2 expression, A549 cells express significantly less ACE2 than WT K562 cells yet internalize heat-inactivated SARS-CoV-2 more efficiently (Figure 4D,E,G–I). Compared to WT K562 cells, SARS-CoV-2 PSV-mediated RFP transduction (at 1 × 10^5^ transducing units) was also significantly higher in WT A549 cells (Figure 4F,G,I), suggesting that ACE2 independent cellular modalities are also involved in the cellular uptake of SARS-CoV-2. Considering A549 cells’ richer expression of SDCs (Figure 4C and Supplementary Figure S9), along with previous findings of increased SARS-CoV-2 uptake due to SDC overexpression, we also explored the involvement of SDCs in SARS-CoV-2 uptake on A549 cells.

**Figure 5:**
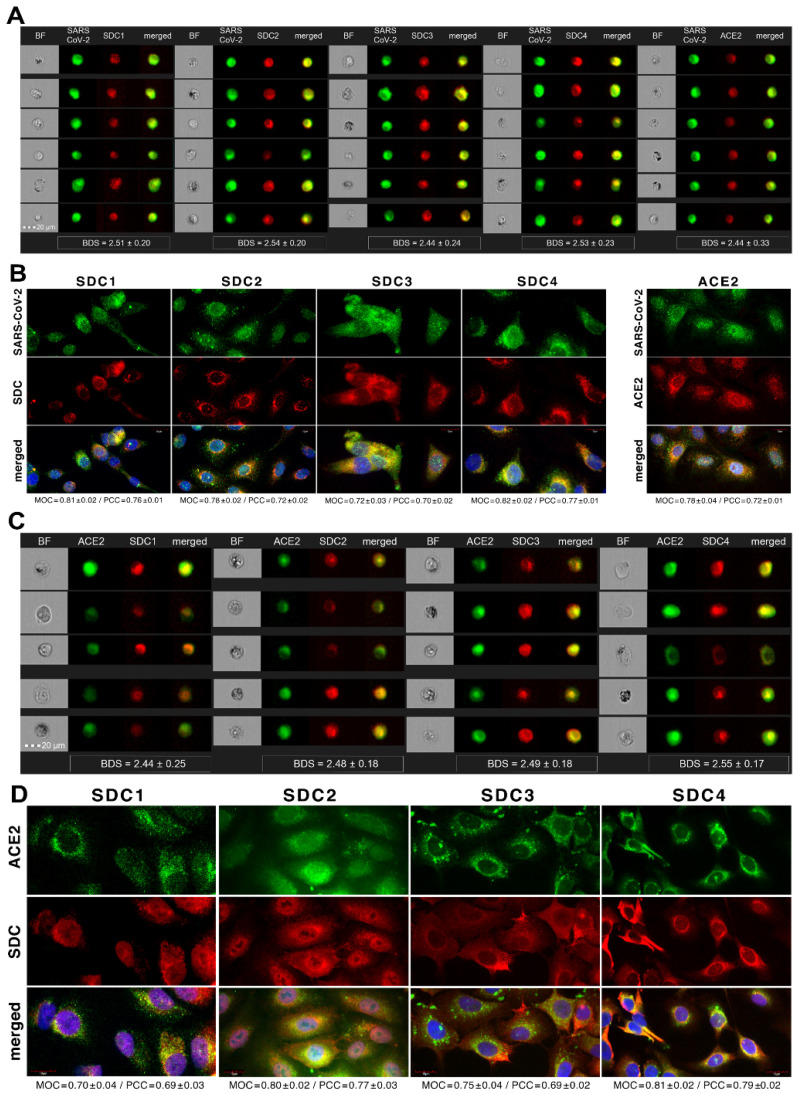
SARS-CoV-2 colocalizes with both SDCs and ACE2 during its uptake in A549 cells. WT A549 cells were incubated with heat-inactivated SARS-CoV-2 (at 1 MOI) for 18 h at 37 °C. After incubation, the cells were treated with, in case of the SARS-CoV-2/SDC colocalization studies, antibodies specific for the spike glycoprotein (along with AF 488-labeled secondary antibody) and APC-labeled SDC antibodies. Colocalization of SARS-CoV-2 and ACE2 was analyzed by using AF 647-labeled antibody against ACE2. For analyzing colocalization between SDCs and ACE2, the SARS-CoV-2-treated cells, after incubation, were treated with AF 488-labeled antibodies against ACE2 and APC-labeled SDC antibodies. Colocalization of SARS-CoV-2 with SDCs and ACE2, or SDCs with ACE2, was then analyzed with imaging flow cytometry and confocal microscopy. (**A**) Imaging flow cytometry visualization of colocalization between SARS-CoV-2 and SDCs and ACE2 in SARS-CoV-2-treated A549 cells. Representative images of four independent experiments are shown. Scale bar = 20 μm. BDS of SARS-CoV-2 and SDCs (or ACE2) represent the mean ± SEM of four independent experiments. Statistical significance between the groups was assessed with ANOVA (no statistically significant differences were detected). (**B**) Confocal microscopy visualization of colocalization between SARS-CoV-2 and SDCs or ACE2 in SARS-CoV-2-treated WT A549 cells. Representative images of four independent experiments are shown. Scale bar = 10 μm. MOC ± SEM and PCC ± SEM for the overlap and colocalization of SARS-CoV-2 with either of the SDC isoforms and ACE2 (indicated below each image) were calculated by analyzing 15 images with ~10 cells in each image (from 3 separate samples). (**C**) Imaging flow cytometry visualization of colocalization between ACE2 and SDCs in SARS-CoV-2-treated WT A549 cells. Representative images of four independent experiments are shown. Scale bar = 20 μm. The indicated BDS of ACE2 and SDCs represent the mean ± SEM of four independent experiments. (**D**) Confocal microscopy visualization of colocalization between ACE2 and SDCs in SARS-CoV-2-treated WT A549 cells. Representative images of four independent experiments are shown. Scale bar = 10 μm. MOC ± SEM and PCC ± SEM for the overlap and colocalization of ACE2 and SDCs (indicated below each image) were calculated by analyzing 15 images with ~10 cells in each image (from 3 separate samples).

Imaging flow cytometry and confocal microscopy analyses demonstrated significant colocalization of SDCs with SARS-CoV-2 (Figure 5A,B). ACE2, the established receptor for SARS-CoV-2, also colocalized with the virus in uptake studies on A549 cells (Figure 5A,B). The next steps showed that ACE2 and SDCs colocalize during SARS-CoV-2 uptake, suggesting that ACE2 and SDCs collaborate in mediating SARS-CoV-2 internalization (Figure 5C,D). Co-IP studies also confirmed SARS-CoV-2 binding SDC4 but also ACE2 (Supplementary Figure S10).

### 2.4. SDCs Facilitate Cellular Uptake of the SARS-CoV-2 Spike Protein S1 Subunit

**Figure 6:**
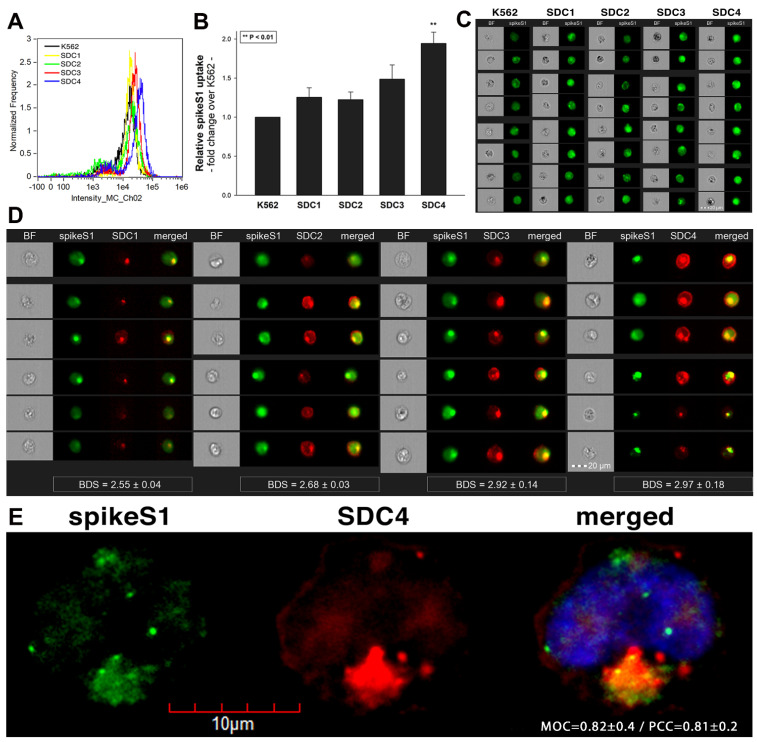
Cellular uptake of spikeS1 into SDC transfectants. WT K562 cells and SDC transfectants were incubated with spikeS1 for 18 h at 37 °C. After incubation, the cells were washed, trypsinized, fixed, permeabilized and treated with FITC-labeled antibodies specific for the N-terminal His-tag of the recombinant spikeS1. Cellular uptake of spikeS1 was then analyzed with imaging flow cytometry and confocal microscopy. (**A**) A representative flow cytometry histogram showing the intracellular fluorescence of spikeS1-treated WT K562 cells and SDC transfectants. (**B**) Detected fluorescence intensities were normalized to spikeS1-treated WT K562 cells as standards. The bars represent the mean ± SEM of three independent experiments. Statistical significance vs. standards was assessed with ANOVA. ** *p* < 0.01. (**C**) BF and fluorescent cellular images of spikeS1-treated WT K562 cells and SDC transfectants. Scale bar = 20 μm. (**D**) Imaging flow cytometry visualization of colocalization between SDCs and spikeS1. Representative images of four independent experiments are shown. Scale bar = 20 μm. The indicated BDS of spikeS1 and SDCs represent the mean ± SEM of four independent experiments. Statistical significance between the groups was assessed with ANOVA. No statistically significant differences were detected. (**E**) Colocalization of spikeS1 and SDC4 detected with confocal microscopy. Representative images of three independent experiments are shown. Scale bar = 10 μm. MOC ± SEM and PCC ± SEM for the overlap and colocalization of SDC4 with spikeS1 (indicated on the image) was calculated by analyzing 12 images with an average of 12 cells in each image (from 3 separate samples).

To widen the understanding of SARS-CoV-2’s complex cellular entry, we also explored the cellular interactions of the SARS-CoV-2 spike protein S1 subunit (spikeS1), responsible for mediating attachment to host cells. At first, we explored the potential cellular uptake of spikeS1 into SDC transfectants created in K562 cells. Just like in the case of heat-inactivated SARS-CoV-2, SDC transfectants with an equal amount of HS expression were selected and, along with WT K562 cells, treated with spikeS1. After 18 h of incubation, cellular uptake was detected by incubating the spikeS1-treated, fixed and permeabilized cells with fluorescently (FITC) labeled antibody specific for the N-terminal His-tag of the recombinant spikeS1. For imaging flow cytometry analyses, extracellular fluorescence of surface-attached spikeS1 was removed with trypsinization (according to the method described by Nakase et al. [71,72]). Imaging flow cytometry analyses revealed increased uptake of spikeS1 into SDC lines (Figure 6A–C). Among SDCs, SDC4 significantly increased the uptake of spikeS1 (*p* < 0.01) (incubating the cells with the fluorescently labeled anti-His tag antibodies without spikeS1 pretreatment did not induce any difference in fluorescence among the applied K562 cells and SDC transfectants, showing that no unspecific binding influenced the detected fluorescence intensities in spikeS1-treated cells (Supplementary Figure S11)). Colocalization studies revealed significant colocalization between spikeS1 and SDC4, suggesting the same route SDC4 and spikeS1 follow during cellular entry (Figure 6D,E). Namely, the BDS, the MOC and the PCC for SDC4 and spikeS1 were around 3 and 0.8, respectively, indicating significant colocalization (Figure 6D,E).

### 2.5. Contribution of Various Parts of the SDC4 Ectodomain to SpikeS1 Uptake

**Figure 7:**
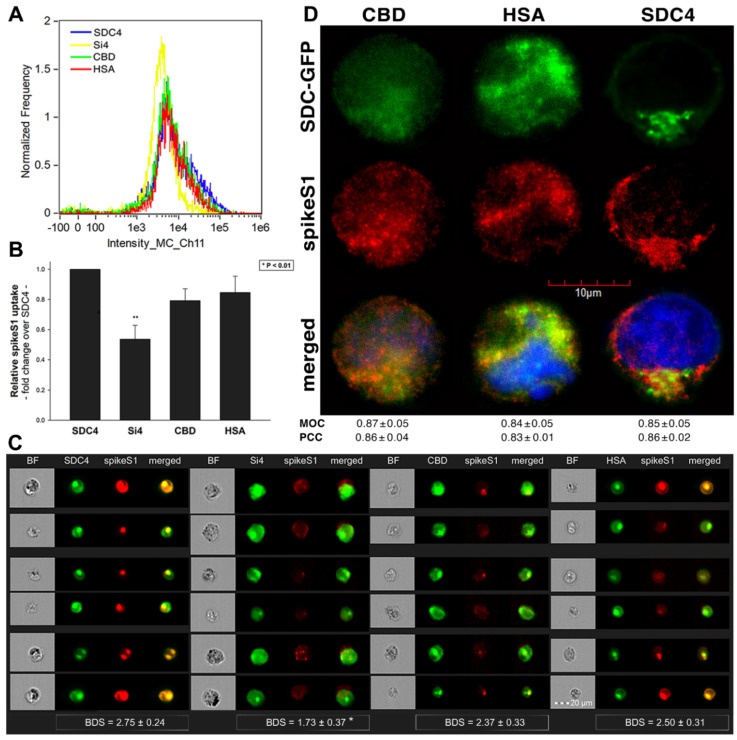
Contribution of the various parts of the SDC4 ectodomain to spikeS1 uptake. SDC4 transfectants and mutants were incubated with spikeS1 for 18 h at 37 °C. After incubation, the cells were washed, trypsinized, fixed, permeabilized and treated with AF 633-labeled antibodies specific for the His-tag of the recombinant spikeS1. Cellular uptake of spikeS1 was then analyzed with imaging flow cytometry and confocal microscopy. (**A**) A representative flow cytometry histogram showing the intracellular fluorescence of spikeS1-treated SDC4 transfectants and mutants. (**B**) Detected fluorescence intensities were normalized to spikeS1-treated WT SDC4 transfectants as standards. The bars represent the mean ± SEM of three independent experiments. Statistical significance vs. standards was assessed with ANOVA. ** *p* < 0.01. (**C**) Imaging flow cytometry visualization of colocalization between SDC4 mutants and spikeS1. Representative images of four independent experiments are shown. Scale bar = 20 μm. The indicated BDS represent the mean ± SEM of four independent experiments. Statistical significance vs. spikeS1-treated SDC4 transfectants was assessed with ANOVA. * *p* < 0.05. (**D**) Colocalization of spikeS1 and SDC4 mutants detected with confocal microscopy. Representative images of three independent experiments are shown. Scale bar = 10 μm. MOC ± SEM and PCC ± SEM fwas calculated by analyzing 12 images with an average of 12 cells in each image (from 3 separate samples). No statistically significant differences were detected between the groups.

As both SARS-CoV-2 and spikeS1 demonstrated similarly increased internalization into SDC transfectants, suggesting that spikeS1 would be a key modality to facilitate SARS-CoV-2’s interactions with SDCs, we also explored the interaction of spikeS1 with SDC4 structural mutants (Figure 3A). Transfectants of GFP-tagged Si4, CBD, HSA and SDC4 were incubated with spikeS1 for 18 h. After incubation, the cells were fixed, permeabilized and treated with AF 647-labeled secondary antibodies specific for the N-terminal His-tag of spikeS1. Fluorescence was then analyzed with imaging flow cytometry and confocal microscopy. To remove extracellularly attached spikeS1, the trypsinization method of Nakase et al. was applied [71,72]. Just like in the case of SARS-CoV-2, both the HSA and CBD proved to serve a significant role in interacting with spikeS1. Namely, deleting both the CBD and the HSA (with HS chains) significantly reduced cellular uptake of spikeS1, as shown by the markedly reduced intracellular fluorescence detected on Si4 mutants (Figure 7A–D). However, the insignificant reduction in the cellular fluorescence of spikeS1-treated CBD or HSA mutants showed that deleting either the CBD or the HS chains could not reduce the internalization of spikeS1 significantly (Figure 7B,C). Thus, the CBD or the HSA site of SDC4 could compensate for removing either the HS chains or the CBD, respectively. In the SDC4 transfectants and the HSA and CBD mutants, the BDS score of colocalization between the fluorescent signals of the SDC4 constructs and spikeS1 also showed a high degree of colocalization (Figure 7C). Compared to SDC4 transfectants, the BDS score of Si4 mutants lacking HS chains and CBD were significantly reduced (*p* < 0.05). Microscopic colocalization also showed substantial colocalization of the spikeS1 with either of the HSA or CBD mutants, with MOC and PCC values around 0.8, demonstrating that spikeS1 could attach to both the HS chains or the CBD of SDC4 (Figure 7D).

Co-IP studies also confirmed the ability of the CBD or the HS chains to bind spikeS1 (Supplementary Figure S12). Our studies with the SDC4 deletion mutants thus revealed that besides interacting with the polyanionic HS chains, spikeS1 also interacts with the CBD of SDC4 (incubating the cells with the fluorescently labeled anti-His tag antibodies without spikeS1 pretreatment did not induce any difference in fluorescence among the applied SDC4 transfectants and SDC4 mutants, showing that no unspecific binding influenced the detected fluorescence intensities in spikeS1-treated cells (Supplementary Figure S13)).

### 2.6. Interaction of SpikeS1 with SDC4 in A549 Cells

**Figure 8:**
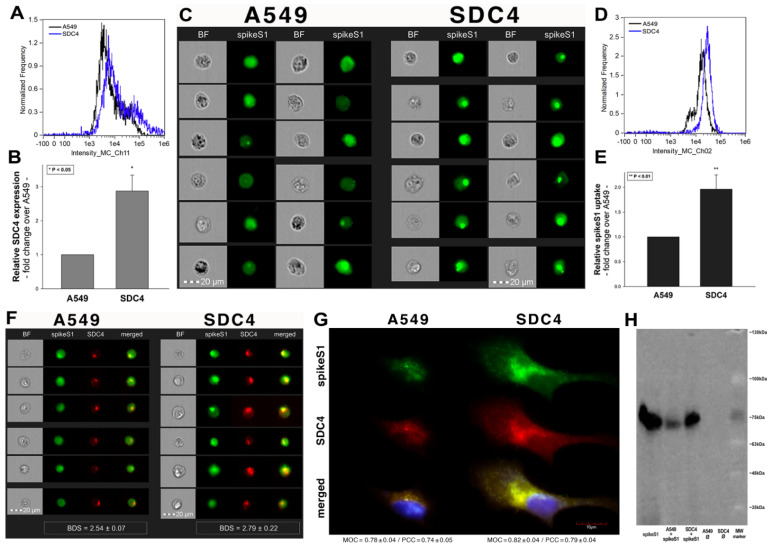
SDC4 overexpression increases spikeS1 uptake in A549 cells. WT A549 cells and SDC4 transfectants (created in A549 cells) were incubated with recombinant spikeS1 for 18 h at 37 °C. After incubation, spikeS1-treated cells were trypsinized, fixed, permeabilized and treated with fluorescently labeled anti-His tag antibodies. After antibody treatment, intracellular fluorescence was analyzed with either imaging flow cytometry or confocal microscopy. (**A**,**B**) SDC4 expression measured with flow cytometry by using APC-labeled SDC4 antibodies. (**A**) Representative flow cytometry histograms showing SDC4 expression levels of WT A549 cells and SDC4 transfectants. (**B**) Detected SDC4 expression levels were normalized to those of WT A549 cells as standards. The bars represent the mean ± SEM of three independent experiments. Statistical significance vs. standards was assessed with ANOVA. * *p* < 0.05. (**C**) Cellular images representing intracellular fluorescence of spikeS1-treated WT A549 cells and SDC4 transfectants. (**D**,**E**) SDC4 overexpression increases spikeS1 uptake. (**D**) Representative flow cytometry histograms showing the intracellular fluorescence of spikeS1-treated WT A549 cells and SDC4 transfectants. (**E**) Fold change in spikeS1 uptake following SDC4 overexpression. The bars represent the mean ± SEM of three independent experiments. Statistical significance vs. spikeS1-treated WT A549 cells as standards was assessed with ANOVA. ** *p* < 0.01. (**F**) Colocalization of spikeS1 in WT A549 cells and SDC4 transfectants as detected with imaging flow cytometry. The indicated BDS between spikeS1 and SDC4 represents the mean ± SEM of three independent experiments. (**G**) Confocal microscopy visualization of colocalization between spikeS1 and SDC4 in WT A549 cells and SDC4 transfectants. Representative images of three independent experiments are shown. Scale bar = 10 μm. The MOC ± SEM and PCC ± SEM for the overlap and colocalization of SDC4 with spikeS1 are indicated in the images. The MOC and PCC values were calculated by analyzing 12 images with an average of 12 cells in each image (from 3 separate samples). (**H**) A representative Western blot showing spikeS1 immunoprecipitated with SDC4 in WT A549 cells and SDC4 transfectants. Lane 1: 0.5 µg of spikeS1; lanes 2–3: immunoprecipitates of spikeS1-treated WT A549 cells and SDC4 transfectants, respectively; lanes 4–5: immunoprecipitate of WT A549 cells and SDC4 transfectants untreated with spikeS1 (controls). Standard protein size markers are indicated on the right.

Previous studies showed modest yet detectable SDC4 expression levels in A549 cells (Figure 4A–C). As SDC4 demonstrated the highest uptake efficacy of spikeS1, we created an SDC4 transfectant exhibiting elevated SDC4 expression (Figure 8A,B). It is worth noting that SDC4 overexpression did not affect the modest ACE2 expression in A549 cells (Supplementary Figure S14). Increased SDC4 expression, with unaffected ACE2 levels, resulted in increased cellular uptake of spikeS1 (Figure 8C–G). Namely, overexpression of SDC4 increased spikeS1 entry from a low level of WT A549 cells by almost twofold (Figure 8C–E). Colocalization studies revealed that spikeS1 colocalizes with SDC4 during increased spikeS1 entry (as shown by the high BDS, MOC and PCC scores obtained with imaging flow cytometry and confocal microscopy (see details in Figure 8F,G), while co-immunoprecipitation showed increased binding of spikeS1 to SDC4 due to SDC4 overexpression (Figure 8H) (incubating the cells with the fluorescently labeled anti-His tag antibodies without spikeS1 pretreatment did not induce any difference in fluorescence among the applied A549 cell line and SDC4 transfectants, showing that no unspecific binding influenced the detected fluorescence intensities in spikeS1-treated cells (Supplementary Figure S15)).

### 2.7. ACE2 and SDC4 Inhibition Support the Complexity of SARS-CoV-2 Uptake

**Figure 9:**
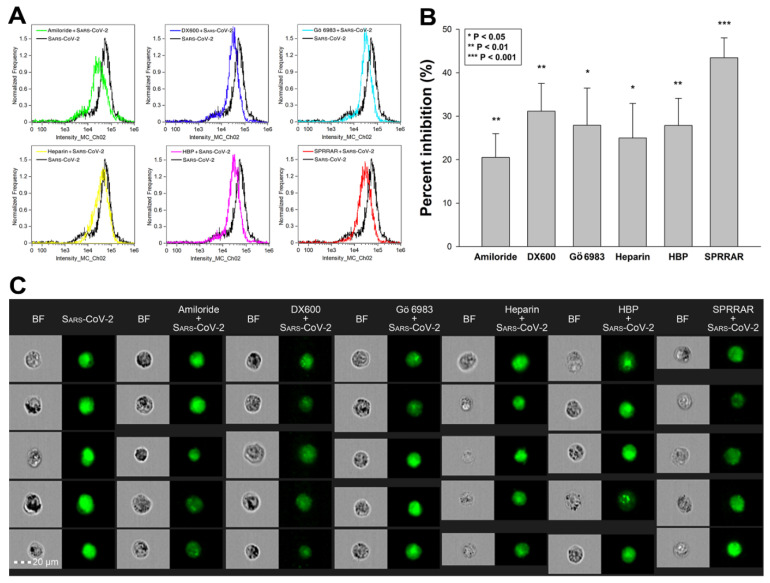
Effects of ACE2 or SDC4 inhibition on SARS-CoV-2 uptake in A549 cells. (**A**) Cellular images of SARS-CoV-2-treated WT A549 cells preincubated with or without either of the inhibitors: amiloride, DX600, Gö 6983, heparin, HBP and SPRRAR. After 18 h of incubation, the cells were washed, trypsinized, fixed, permeabilized and treated with antibodies specific for the spike glycoprotein (along with AF 488-labeled secondary antibody). Intracellular fluorescence was then analyzed with imaging flow cytometry. (**B**) Flow cytometry histograms representing intracellular fluorescence of SARS-CoV-2-treated WT A549 cells preincubated with or without any inhibitors. (**C**) The effect of an inhibitor was expressed as percent inhibition, calculated with the following formula: [(X − Y)/X] × 100, where X is the fluorescence intensity obtained on cells treated with SARS-CoV-2 in the absence of the inhibitor, and Y is the fluorescence intensity obtained on cells treated with SARS-CoV-2 in the presence of the inhibitor. The bars represent the mean ± SEM of four independent experiments. Statistical significance vs. controls treated with SARS-CoV-2 in the absence of the inhibitor was assessed with ANOVA. * *p* < 0.05; ** *p* < 0.01; *** *p* < 0.001.

Utilizing SDC transfectants and A549 cells, we managed to reveal an interplay of ACE2 and SDCs in mediating the cellular uptake of SARS-CoV-2. Developing efficient SARS-CoV-2 therapeutics requires the consideration of the complexity of SARS-CoV-2’s cellular interplay. Our studies also demonstrated this complexity with representative inhibitors of various cellular pathways. The following inhibitors were applied: amiloride hydrochloride (amiloride) as the well-established inhibitor of micropinocytosis [75]; DX600 as a selective ACE2 blocker [76]; Gö 6983 as a selective PKC antagonist [77,78,79]; heparin as the inhibitor of electrostatic interactions of GAGs [80]; a heparin-binding peptide (WQPPRARI, abbreviated as HBP) derived from fibronectin [81,82,83]; and a small peptide (SPRRAR) derived from the heparin-binding motif of SARS-CoV-2. Among them, amiloride and heparin are considered as more general inhibitors, DX600 and Gö 6983 are selective. As PKC activation is required for triggering SDC-mediated uptake, the application of Gö 6983 served the exploration of SDCs in SARS-CoV-2 internalization. The HBP (WQPPRARI) from fibronectin competes to the attachment of HS chains of SDCs, while SPRRAR derived from spikeS1 contains a very efficient heparin-binding motif. As shown in Figure 9A–C, uptake studies demonstrated that while all of the applied inhibitors efficiently reduced SARS-CoV-2 uptake, SPRRAR, a peptide derived from the spikeS1 of SARS-CoV-2 emerged as the most potent one, demonstrating that molecularly detailed understanding of the SARS-CoV-2 internalization could indeed lead to the rational development of potent SARS-CoV-2 therapeutic leads (preincubating the cells with the inhibitors did not influence cell viability, demonstrating that the reduced SARS-CoV-2 uptake due to inhibitor treatment did not arise from disturbed cellular viability (Supplementary Figure S16)).

**Figure 10:**
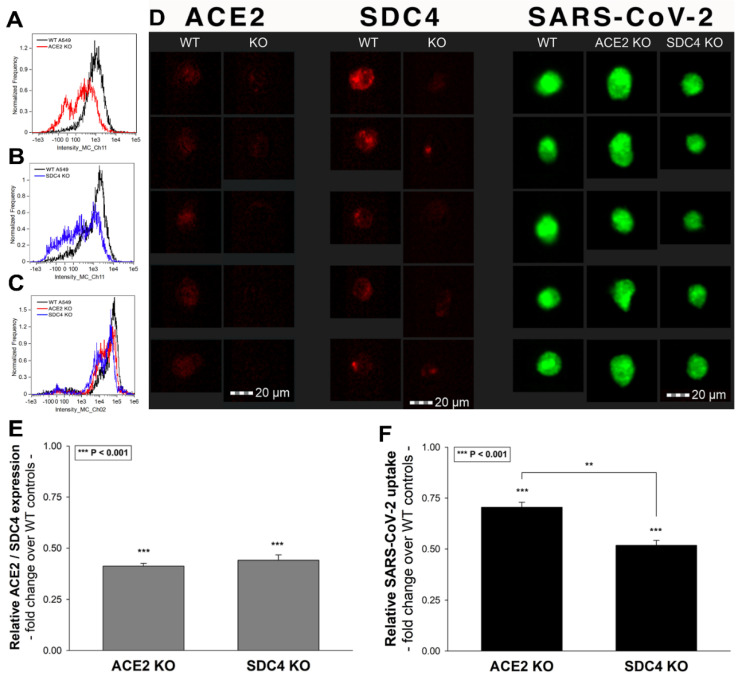
Effect of ACE2 or SDC4 knockdown on SARS-CoV-2 cellular entry. SDC4 and ACE2 knockdown in A549 cells was performed using a lentiviral vector system specific to human ACE2 and SDC4 shRNA. Stable KO cells were then selected, and along with WT A549 cells, were incubated with heat-inactivated SARS-CoV-2 (at 1 MOI) for 18 h at 37 °C. (**A**,**B**) Representative flow cytometry histograms showing ACE2 and SDC4 expression levels of WT A549, ACE2 (**A**) and SDC4 KO (**B**) cells. (**C**) Representative flow cytometry histograms showing the intracellular fluorescence of SARS-CoV-2-treated WT A549, ACE2 KO and SDC4 KO cells. (**D**) Imaging flow cytometry visualization of ACE2 and SDC4 expression, along with SARS-CoV-2 internalization of WT A549, ACE2 KO and SDC4 KO cells. Representative images of four independent experiments are shown. Scale bar = 20 μm. (**E**) Relative ACE2 and SDC4 expression levels of ACE2 KO and SDC4 KO cells. Detected ACE2 and SDC4 expression levels were normalized to those of WT A549 cells as standards. The bars represent the mean ± SEM of three independent experiments. Statistical significance vs. standards was assessed with analysis of variance (ANOVA). *** *p* < 0.001. (**F**) Detected intracellular fluorescence intensities of SARS-CoV-2-treated ACE2 and SDC4 KO cells were normalized to SARS-CoV-2-treated WT A549 cells as standards. The bars represent the mean ± SEM of three independent experiments. Statistical significance vs. standards (i.e., SARS-CoV-2-treated WT A549 cells) and between ACE2 and SDC4 KO cells were assessed with ANOVA. ** *p* < 0.01; *** *p* < 0.001.

To further explore the contribution of ACE2 and SDC4 to SARS-CoV-2 uptake, SDC4 and ACE2 knockdown in A549 cells was performed using ACE2- and SDC4-specific human shRNA lentiviral vectors (Figure 10A,B,D,E and Supplementary Figure S17). Compared to WT A549 cells, SARS-CoV-2 treatment of ACE2 and SDC4 KO cells resulted in significantly reduced virus uptake (Figure 10C,D,F). Compared to ACE2 knockdown, the effect of SDC4 KO exerted a significantly (*p* < 0.01) more pronounced inhibitory effect on SARS-CoV-2 internalization (Figure 10D,F), suggesting a substantial role is played by SDC4 in SARS-CoV-2 uptake.

## 3. Discussion

The current outbreak of SARS-CoV-2, a novel coronavirus with a yet undetermined origin, causes an unprecedented threat to modern societies [5,84,85]. Due to the fact that the WHO declared SARS-CoV-2 a pandemic, intense research is manifested to deliver specific medicines halting virus spread and infection [12]. Pharmaceutical efforts in delivering efficient yet safe medicines against SARS-CoV-2 are being hampered by the unknown details of SARS-CoV-2 cellular biology [10,86]. While most studies on SARS-CoV-2 emphasize the difference between SARS-CoV-2 and SARS-CoV, several findings on SARS-CoV are also being regarded as applicable for SARS-CoV-2 [87]. One such fundamental finding on SARS-CoV that has been widely accepted for SARS-CoV-2 is ACE2 serving as the primary yet sole cellular entry receptor, facilitating virus internalization after transmembrane protease serine type 2 (TMPRSS2)-mediated cleavage of the spike protein [27,28,88,89]. However, the failure of highly specific ACE2 pharmaceuticals to stop SARS-CoV-2 infection and its related disease, COVID-19, clearly highlights the complexity of SARS-CoV-2 internalization [90]. Emerging evidence shows the collaboration of ACE2 and HSPGs during SARS-CoV-2 uptake [37].

It has been widely accepted that cell surface HSPGs provide efficient cellular entry for many pathogens [50,51,52]. SDCs are the only transmembrane family of HSPGs [38,39]. Due to their versatile and polyanionic HS chains, SDCs bind a myriad of extracellular ligands, including several viruses. Due to their evolutionary conserved intracellular PDZ domains, SDCs also interact with a whole range of intracellular signaling molecules, thus providing a transmembrane link between extracellular HS-mediated processes and intracellular signaling cascades [40,42,47,91]. Attachment to HS chains of HSPGs and interaction with PDZ domains has been already explored for SARS-CoV, but not yet investigated for SARS-CoV-2 [29,30,32]. However, recent studies explored the interaction of the SARS-CoV-2 with heparin [92,93]. According to these studies, the conformation change induced by the attachment of spikeS1 to heparin is required for efficient SARS-CoV-2 entry into the cells [93]. Another very recent finding also showed heparin effectively blocking SARS-CoV-2 invasion into Vero cells [94]. It has to be noted that the primary sequence of spikeS1 (namely Pro681-Arg685) contains the heparin-binding core motif PRRAR [33,34,35] (Supplementary Figure S1). According to current reports, this heparin-binding motif may facilitate SARS-CoV-2 host cell entry [36].

Considering scientific evidence supporting the involvement of HSPGs in SARS-CoV-2 infection, we set up a study exploring the contribution of SDCs to the cellular uptake of SARS-CoV-2 and spikeS1, the subunit responsible for cell attachment of the virus. Up till now, this is the first study exploring the specific involvement of the whole SDC family in the cellular uptake of SARS-CoV-2. According to our results, the overexpression of SDCs, including SDC4, the isoform most abundant in the lung, significantly increases cellular uptake of SARS-CoV-2. Entry via SDCs enabled efficient gene transduction with the SARS-CoV-2 pseudovirus (PSV), implying that the SDC-mediated internalization pathway maintains the viral particles’ biological activity. Thus, SDCs have a crucial involvement in facilitating the cellular entry of SARS-CoV-2, while the spikeS1 plays a significant role in the interactions with SDCs. The HS chains do not exclusively drive the binding of SARS-CoV-2 to the SDC4 ectodomain but is also influenced by other parts of the SDC ectodomain, including SDC4’s CBD. In our studies with SDC4 deletion mutants, the CBD mutants lacking HS chains exhibited internalization characteristics comparable to WT SDC4 transfectants, thus supporting the SDC core protein’s involvement in the interaction with the virus. SDC4’s CBD also played a dominant role in spikeS1–SDC4 interactions, emphasizing the need to go beyond standard HS–virus interactions in understanding the molecular interplay between SARS-CoV-2 and SDCs. In our studies, heparin, a polyanionic agent effectively inhibiting the attachment of several viruses to polyanionic HS on cell surface proteoglycans [52], even at very high doses, did not emerge as the most potent blocker of virus uptake. At the same time, SPRRAR, a heparin-binding peptide derived from spikeS1, showed superior efficacy to block virus uptake.

It is worth noting that proteoglycans’ sulfation pattern, contributing significantly to proteoglycan HS chains’ structural diversity, defines the binding of ligands [38,45,48,95,96,97]. Although most cells express more than one HSPG at their cell surface, several lines of evidence indicate that HS chains attached to different core proteins on the same cell surface have the same sulfation patterns [39,98,99,100]. The detected difference in the cellular uptake of SARS-CoV-2 (and spikeS1) between various SDC transfectants expressing a similar level of HS also indicates that interaction of SARS-CoV-2 with SDCs is also influenced by HS independent parts of the SDC core protein. Investigating the cellular uptake of SARS-CoV-2 in stable SDC transfectants with an equal amount of HS expression was, therefore, critical in understanding the influence of the core protein (i.e., the non-GAG parts) on interactions with the virus. As the fine structure and ligand binding of SDCs’ HS chains show cell type-specific differences [100,101,102], the involvement of different cell lines in the analyses of SDCs interaction with SARS-CoV-2 was also crucial for exploring the virus’ cellular interactions.

Examination of SARS-CoV-2’s internalization in A549 cells also revealed the interplay of SDCs and ACE2, where both modalities (i.e., ACE2 and SDCs) interact with SARS-CoV-2 to facilitate its cellular entry. Therefore, our results do not abolish the ACE2 hypothesis but depict a molecularly more diverse scenario involving the collaboration of SDCs and ACE2 in mediating SARS-CoV-2 internalization. The finding that SDC overexpression, with unaffected ACE2 expression levels, results in increased SARS-CoV-2 cellular entry highlights the importance of SDCs in the complex molecular interplay of SARS-CoV-2 internalization. The importance of SDCs was also supported by studies showing that the SDC4 knockdown exerts a significant reduction on SARS-CoV-2 uptake (i.e., even more significant than ACE2).

SDCs have already been regarded as a favorite binding site for several viruses and bacteria [51,52]. In line with these findings, our results suggest that SDCs play a crucial role in facilitating SARS-CoV-2’s cellular entry. Parasites’ preference for SDC modalities also stems from SDCs’ ability to mediate their ligands’ internalization through a pathway avoiding lysosomal degradation [45,46,47,64]. Unlike the classical clathrin-mediated endocytosis driving internalized particles into lysosomes for degradation, viruses entering the cells via SDCs can avoid lysosomal degradation and maintain their virulence [103,104]. The increased gene transduction of the SARS-CoV-2 PSV in SDC overexpressing cell lines highlights the importance of SDCs in SARS-CoV-2 infection. Namely, SDCs, especially SDC4, provide an efficient entry mechanism that prevents SARS-CoV-2 from lysosomal degradation.

Although most abundant in the lungs, the widespread distribution of SDC4 and other SDCs in the human body can explain the ability of the SARS-CoV-2 spread to various organs. Moreover, the increased uptake of SARS-CoV-2 into cells expressing other SDCs, including neuronal SDC3, might imply the virus’s ability to infect the CNS cells. Considering the ability of HIV-1 to exploit SDCs to enter the brain [105], our finding on the increased uptake of SARS-CoV-2 into SDC3 transfectants might explain the molecular determinants of SARS-CoV-2’s potential CNS entry [106]. Moreover, altered expression of SDCs is associated with cardiovascular disorders, diabetes, obesity and Alzheimer’s disease, just some of the most common comorbidities correlating with poorer clinical outcomes in COVID-19 [107,108,109,110,111,112,113,114,115].

Overall, our results obtained in highly specific SDC assays present SDCs as important mediators of SARS-CoV-2 cellular entry and highlight SDCs, including the lung abundant SDC4, as a novel therapeutic target against SARS-CoV-2 infection. Our findings are in line with recent reports suggesting the potential role of HSPGs in the cellular entry of SARS-CoV-2 and explore a molecularly more intricate interplay of ACE2 and SDCs in facilitating SARS-CoV-2 uptake. Inhibitor studies show the efficacy of inhibitors of both pathways (i.e., ACE2 or SDC dependent) in blocking SARS-CoV-2 internalization while highlighting the efficacy of peptide (SPRRAR) derived from the heparin-binding motif of SARS-CoV-2. Pharmaceutical development of efficient medicines against SARS-CoV-2 infection should consider a more complex interplay of potential receptors to develop efficient mono- or combinatorial therapeutic strategies against SARS-CoV-2 infection.

## 4. Materials and Methods

### 4.1. Heat-Inactivated SARS-CoV-2, Pseudovirus, Recombinant Proteins and Peptides

Heat-inactivated SARS-CoV-2 (strain: 2019-nCoV/USA-WA1/2020) was purchased from ATCC (Manassas, VA, USA; cat. no. ATCC VR-1986HK), the N-terminally His-tagged recombinant SARS-CoV-2 spike protein S1 subunit (region Val16–Gln690) from RayBiotech (Peachtree Corners, GA, USA) recombinant human syndecan-4 from R&D Systems (Minneapolis, MN, USA; cat. no. 2918-SD) and recombinant human ACE2 from Abcam (Cambridge, UK; ab151852). Peptide inhibitors utilized for the studies were purchased from either Bachem (Bubendorf, Switzerland; DX600) or Genscript (Leiden, The Netherlands; SPRRAR and WQPPRARI). SARS-CoV-2 pseudovirus-RFP (SARS-CoV-2 PSV-RFP), recombinant pseudotyped lentiviral particles containing SARS-CoV-2 spike protein and encoding red fluorescent protein (RFP) in their lentiviral genome was purchased from GeneMedi (Shanghai, China; cat. no. GM-2019nCoV-PSV01).

### 4.2. SDC Constructs, Cell Culture and Transfection

Full-length SDC1–4 and SDC4 deletion mutants and transfectants, established in K562 cells (ATCC CCL-243), were created as described previously [43,45,46]. No His-tags were applied for the SDC constructs. Stable SDC transfectants were selected by measuring SDC expression with flow cytometry using APC-labeled SDC antibodies specific for the respective SDC isoform (all RnD Systems (Minneapolis, MN, USA); SDC1: monoclonal rat IgG1 Clone #359103, cat. no. FAB2780A [116,117,118]; SDC2: monoclonal rat IgG2B Clone #305515, cat. no. FAB2965A [119,120,121]; SDC3: polyclonal goat IgG, cat. no. FAB3539A [119,122]; SDC4: monoclonal rat IgG2a clone #336304, cat. no. FAB29181A [45,46]). SDC4 overexpressing A549 clones, along with WT A549 cells (ATCC CCL-185), were then grown in advanced MEM medium (Thermo Fischer Scientific, Waltham, MA, USA) supplemented with 10% FCS (Gibco, New York, NY, USA) at 37 °C in a humified 5% CO_2_ containing air environment.

### 4.3. Flow Cytometry Analysis of HS, ACE2 and SDC Expression

HS and SDC expression of applied cell lines (WT K562 and SDC transfectants) were measured with flow cytometry by using anti-human HS antibody (10E4 epitope (Amsbio, Abingdon, UK)), AF 488-labeled anti-mouse IgM and APC-labeled antibody as described previously [45,46]. SDC transfectants with almost equal amounts of HS expression were selected for further uptake studies [45,46]. ACE2 expression of WT K562 and A549 cells, along with SDC transfectants, was measured with human ACE-2 AF 647-conjugated antibody (R&D Systems (Minneapolis, MN, USA), cat. no. FAB9332R) according to the manufacturer’s protocol.

### 4.4. Establishment of ACE2 or SDC4 KO Cell Lines

SDC4 and ACE2 knockdown in A549 cells was performed using a lentiviral vector system specific to human ACE2 and SDC4 shRNA (cat. nos. sc-41400-SH, sc-36588) according to the manufacturer’s protocol (Santa Cruz Biotechnology, Inc., Dallas, TX, USA). Stable KO cells were selected in 2 mg G418 and sorted using imaging flow cytometry (Amnis FlowSight, Luminex Corporation, Austin TX, USA) with APC-conjugated anti-SDC4 and Alexa Fluor 647-labeled anti-ACE2 antibodies. Cellular expression of ACE2 and SDC4 following knockdown was also determined with Western blotting. WT A549 and SDC4 and ACE2 shRNA-treated cells were grown in 24-well plates for 24 h; then, the medium was removed and the cells were washed (with PBS) and lysed in RIPA buffer. Protein concentrations were measured with a spectrophotometer (Metertech (Trondheim, Norway) UV/VIS). Equal amounts of protein from cell lysates were then subjected to SDS-PAGE on 7.5–12.5% gradient gels and electroblotted onto PVDF membranes using the Mini Wide Vertical Electrophoresis gel system (Cleaver Scientific, Rugby, UK). The membranes were blocked in TBST with 5% dry milk, washed, incubated with anti-SDC4 antibody (Santa Cruz Biotechnology (Dallas, TX, USA), Inc.; cat. no. sc-12766) and anti-ACE2 antibody (Abcam (Cambridge, UK); cat. no. ab151852) diluted in TBST with 1% dry milk for 2 h, and then incubated with HRP-conjugated secondary antibodies (anti-mouse IgG-HRP, Invitrogen (Carlsbad, CA, USA; cat. no. 31450); anti-rabbit IgG-HRP, Abcam (Cambridge, UK; cat. no. ab97051)). Chemiluminescence detection reagent (Luminata Crescendo Western Blotting HRP Reagents) was used for protein visualization, and the signal was detected with UVITEC Alliance Q9 Advanced imager. β-Tubulin (mouse monoclonal, Santa Cruz Biotechnology (Dallas, TX, USA), Inc., cat. no. sc-5274) was used as loading control.

### 4.5. Pseudovirus Studies

WT K562 and A549 cells, along with SDC transfectants (created in K562 cells), were seeded in 24-well plates with 1 × 10^5^ cells/well. After 24 h of culture, cells were treated with 1 × 10^5^ transducing units of SARS-CoV-2 PSV-RFP according to the manufacturer’s instructions (GeneMedi, Shanghai, China). After 72 h of incubation, RFP expression of SARS-CoV-2 PSV-treated cells was assessed with imaging flow cytometry (Amnis FlowSight, Luminex, Austin, TX, USA).

### 4.6. Flow Cytometry Analysis of SARS-CoV-2 and SpikeS1 Uptake

WT K562 and A549 cells, SDC transfectants and SDC4 structural mutants were utilized to quantify the internalization of spikeS1. Briefly, 6 × 10^5^ cells/mL in DMEM/F12 medium were incubated with either SARS-CoV-2 (at 1 MOI) or spikeS1 (at a concentration of 2 μg/mL) for 18 h at 37 °C. After 18 h of incubation, the cells were trypsinized (with the method described by Nakase et al. [71,72]) to remove the extracellularly attached virus particles or spikeS1 from the cell surface. The cells were then washed, fixed, permeabilized, and blocked with the appropriate serum for 1 h at room temperature. In the case of spikeS1, the cells were then treated with fluorescently (either FITC or in the case of SDC4 mutants, AF 647) labeled anti-His tag antibodies (rabbit poly and monoclonal, Abcam (Cambridge, UK), cat. no. ab1206 and ab237337) for 1 h. In the case of SARS-CoV-2, the cells were then treated with mouse monoclonal (1A9) to SARS spike glycoprotein (Abcam (Cambridge, UK), cat. no. 273433), followed by treatment with either AF 488- (SDC transfectants and A549 cells) or AF 633-labeled (SDC4 mutants) goat anti-mouse IgG (both Invitrogen (Carlsbad, CA, USA), cat. no. A-11001 and A-21052, respectively). For the colocalization studies, SDCs made visible by incubating the cells with either APC-labeled SDC or AF-labeled antibodies (1:100), while ACE2 was detected by using either AF 647-labeled human ACE2 antibody (R&D Systems (Minneapolis, MN, USA), cat. no. FAB9332R) or rabbit polyclonal ACE2 antibody (Abcam (Cambridge, UK), cat. no. ab272690) and fluorescently (FITC) labeled anti-rabbit antibody (Sigma, St. Louis, MO, USA). The samples were then rinsed three times with PBS containing 1% BSA and 0.1% Triton X-100 and progressed towards flow cytometry. Cellular uptake was then measured by flow cytometry using an Amnis FlowSight imaging flow cytometer (Amnis Corporation, Seattle, WA, USA). A minimum of 10,000 events per sample was analyzed. Appropriate gating in a forward-scatter-against-side-scatter plot was utilized to exclude cellular debris and aggregates. Fluorescence analysis was conducted with the Amnis IDEAS analysis software. To examine the influence of the fluorescently labeled anti-His tag antibodies or secondary IgGs, some of the cells were also treated with either anti-His tag or secondary antibodies without preincubation with spikeS1 or SARS-CoV-2, and cellular fluorescence was measured with imaging flow cytometry.

### 4.7. Inhibitor Studies

To reveal the involvement of various endocytosis pathways, A549 cells were preincubated with the following inhibitors 30 min before SARS-CoV-2 admission: amiloride hydrochloride (at a concentration of 100 µM; Sigma, St. Louis, MO, USA); DX600 (10 µM; Bachem, Bubendorf, Switzerland); Gö 6983 (10 µM; Sigma, St. Louis, MO, USA); heparin (200 ug/mL; Sigma, St. Louis, MO, USA); a heparin-binding peptide (WQPPRARI, 100 µM; Genscript, Leiden, The Netherlands); and SPRRAR derived from spikeS1 (100 µM; Genscript, Leiden, The Netherlands). After incubation with these inhibitors, the cells were then treated with SARS-CoV-2 and processed for the flow cytometric analyses as described above.

### 4.8. Cell Viability Measurements

The effect of the applied inhibitors was assessed by incubating A549 cells with either of the following inhibitors: amiloride hydrochloride (amiloride, at a concentration of 100 µM); DX600 (10 µM); Gö 6983 (10 µM, Sigma); heparin (200 ug/mL); and a heparin-binding peptide (WQPPRARI) and SPRRAR (100 µM). Eighteen hours after incubation with these inhibitors, the viability of inhibitor-treated WT A549 cells, along with untreated controls, were assessed with the EZ4U cell proliferation assay (Biomedica Gmbh (Vienna, Austria), cat. no. BI-5000) according to the instructions of the manufacturer. Absorbance was measured with a BioTek Cytation 3 multimode microplate reader.

### 4.9. Microscopic Visualization of Uptake

The internalization of SARS-CoV-2 or spikeS1 was visualized by confocal microscopy. WT A549 and WT K562 cells, along with SDC transfectants and SDC4 mutants, were grown on poly-D-lysine-coated glass-bottom 35 mm culture dishes (MatTek Corp. Ashland, MA, USA). After 24 h, the cells were preincubated in DMEM/F12 medium at 37 °C for 30 min before incubation with the spikeS1 at a concentration of 2 μg/mL. After incubation with either SARS-CoV-2 (1MOI) or spikeS1, the cells were rinsed two times with ice-cold PBS, fixed in 4% paraformaldehyde (Sigma, St. Louis, MO, USA), the cell membranes were permeabilized (0.1% Triton X-100) and the cells were blocked with the appropriate serum for 1 h at room temperature, followed by the specific antibody treatments as described for the flow cytometry analyses. The samples were then rinsed three times with PBS containing 0.1% Triton X-100, then stained with DAPI (1:5000) for 5 min, washed three times with PBS and embedded in Fluoromount G (Southern Biotech, Birmingham, AL, USA) [45,46]. The fluorescence distribution was then analyzed on a Leica DMi8 microscope equipped with an Aurox Clarity laser free confocal unit. Sections presented were taken approximately at the mid-height level of the cells. Photomultiplier gain and illumination power were identical within each experiment. The Aurox Visionary software was used for image acquisition by confocal microscopy. For colocalization analyses, the images were analyzed with ImageJ’s (NIH, Bethesda, MD, USA) JACoP plugin [123].

### 4.10. Co-Immunoprecipitation Experiments

SDC4 transfectants or WT K562 or A549 cells, incubated with or without SARS-CoV2 or spikeS1, were processed for co-immunoprecipitation experiments as described previously [45,46]. Briefly, after incubation, the cells were washed twice with ice-cold PBS and treated with cold Pierce IP lysis buffer. The cells were then scraped off to clean Eppendorf tubes, put on a low-speed rotating shaker for 15 min, and centrifuged at 14,000 g for 15 min at 4 °C. The supernatant was then transferred to new tubes and combined with 5 µg of either mouse monoclonal (1A9) SARS-Cov2 spike glycoprotein S1 antibody (Abcam (Cambridge, UK), cat. no. ab273433) or, in the case of the GFP-tagged SDC4 mutants, GFP antibody (Abcam (Cambridge, UK), cat. no. ab6662). The antigen sample/SDC/SARS-CoV-2 or GFP antibody mixture was then incubated overnight at 4 °C with mixing. The antigen sample/antibody mixture was then added to a 1.5 mL microcentrifuge tube containing pre-washed Pierce Protein A/G Magnetic Beads (Thermo Fisher Scientific, Waltham, MA, USA). After incubation at room temperature for 1 h with mixing, the beads were then collected with a MagJET Separation Rack magnetic stand (Thermo Fisher Scientific, Waltham, MA, USA), and supernatants were discarded. To elute the antigen, 100 µL of SDS-PAGE reducing sample buffer was then added to the tubes and samples were heated at 96 °C for 10 min in 1% SDS, and the samples were proceeded to SDS-PAGE [45,46]. The samples were then immunoblotted onto PVDF membranes, and the proteins were detected with specific antibodies as described above. Image acquisition was conducted with UVITEC Alliance Q9 Advanced imaging platform.

### 4.11. Statistical Analysis

Results are expressed as means ± standard error of the mean (SEM). Differences between experimental groups were evaluated by using one-way analysis of variance (ANOVA). Values of *p* < 0.05 were accepted as significant [45,46]. During imaging flow cytometry, the Bright Detail Similarity (BDS) feature of the Amnis IDEAS software was used to measure colocalization between two signals [73]. A BDS score of 2 or greater represents a high degree of overlap. For microscopic colocalization analyses, the Mander’s overlap and Pearson correlation coefficients (MOC and PCC, respectively) were calculated using ImageJ’s JACOP plugin [123] as described by Wesen et al. [124].

## Data Availability

Data are contained within the article or supplementary material.

## Supplementary Materials

The following are available online at https://www.mdpi.com/article/10.3390/ijms22105336/s1. Supplementary Figure S1: The primary sequence of recombinant spike S1 subunit applied in our studies; Supplementary Figure S2: Relative HS expression of SDC transfectants; Supplementary Figure S3: Relative ACE2 expression of K562 cells and SDC transfectants; Supplementary Figure S4: Cellular fluorescence of WT K562 cells and SDC transfectants after AF 488-labeled secondary antibody treatment; Supplementary Figure S5: Colocalization of SARS-CoV-2 with SDCs visualized with confocal microscopy; Supplementary Figure S6: Relative ACE2 expression of SDC4 mutants. ACE2 expression of SDC4 mutants was measured with flow cytometry using AF 647-labeled ACE2 antibody; Supplementary Figure S7: Contribution of the various parts of the SDC4 ectodomain to SARS-CoV-2 uptake; Supplementary Figure S8: Cellular fluorescence of SDC4 mutants after secondary antibody treatment; Supplementary Figure S9: Representative flow cytometry histograms showing the expression levels of SDC isoforms in WT A549 cells; Supplementary Figure S10: SDC4 and ACE2 immunoprecipitated with SARS-CoV-2; Supplementary Figure S11: Control studies with anti-His tag antibody-treated WT K562 cells and SDC transfectants, Supplementary Figure S12: SpikeS1 immunoprecipitated with SDC4 and its mutants; Supplementary Figure S13: Control studies with anti-His tag antibody-treated SDC4 transfectants and mutants; Supplementary Figure S14: Relative ACE2 expression of A549 cells and SDC4 transfectants; Supplementary Figure S15: Control studies with anti-His tag antibody-treated WT A549 cells and SDC4 transfectants (created in A549 cells); Supplementary Figure S16: Cellular viability measurements in the presence of the applied inhibitors; Supplementary Figure S17: Western blot validation of ACE2 and SDC4 knockdown in A549 cells.
