# Supplementary material for: Contribution of Syndecans to the Cellular Entry of SARS-CoV-2
Source: Int J Mol Sci. 2021 May 19;22(10):5336. doi: 10.3390/ijms22105336 (PMC8159090; doi:10.3390/ijms22105336)
Supplement: Supplementary file 1 [file ijms-22-05336-s001.zip › ijms-1213262-supplementary.pdf]

Supplementary Materials: Contribution of syndecans to the cellular entry of SARS-CoV-2

Anett Hudák [1], Annamária Letoha [2], László Szilák [1,2], and Tamás Letoha [1,*]

	20	30	40	50
	**VNLTT**	**RTQLPPAYTN**	**SFTRGVYYPD**	**KVFRSSVLHS**
60	70	80	90	100
**TQDLFLPFFS**	**NVTWFHAIHV**	**SGTNGTKRFD**	**NPVLPFNDGV**	**YFASTEKSNI**
110	120	130	140	150
**IRGWIFGTTL**	**DSKTQSLLIV**	**NNATNVVIKV**	**CEFQFCNDPF**	**LGVYYHKNNK**
160	170	180	190	200
**SWMESEFRVY**	**SSANNCTFEY**	**VSQPFLMDLE**	**GKQGNFKNLR**	**EFVFKNIDGY**
210	220	230	240	250
**FKIYSKHTPI**	**NLVRDLPQGF**	**SALEPLVDLP**	**IGINITRFQT**	**LLALHRSYLT**
260	270	280	290	300
**PGDSSSGWTA**	**GAAAYYVGYL**	**QPRTFLLKYN**	**ENGTITDAVD**	**CALDPLSETK**
310	320	330	340	350
**CTLKSFTVEK**	**GIYQTSNFRV**	**QPTESIVRFP**	**NITNLCPFGE**	**VFNATRFASV**
360	370	380	390	400
**YAWNRKRISN**	**CVADYSVLYN**	**SASFSTFKCY**	**GVSPTKLNDL**	**CFTNVYADSF**
410	420	430	440	450
**VIRGDEVRQI**	**APGQTGKIAD**	**YNYKLPDDFT**	**GCVIAWNSNN**	**LDSKVGGNYN**
460	470	480	490	500
**YLYRLFRKSN**	**LKPFERDIST**	**EIYQAGSTPC**	**NGVEGFNCYF**	**PLQSYGFQPT**
510	520	530	540	550
**NGVGYQPYRV**	**VVLSFELLHA**	**PATVCGPKKS**	**TNLVKNKCVN**	**FNFNGLTGTG**
560	570	580	590	600
**VLTESNKKFL**	**PFQQFGRDIA**	**DTTDAVRDPQ**	**TLEILDITPC**	**SFGGVSVITP**
610	620	630	640	650
**GTNTSNQVAV**	**LYQDVNCTEV**	**PVAIHADQLT**	**PTWRVYSTGS**	**NVFQTRAGCL**
660	670	680	690	700
**IGAEHVNNSY**	**ECDIPIGAGI**	**CASYQTQTNS**	***PRRAR*SVASQ**	

Figure S1. The primary sequence of recombinant spike S1 subunit applied in our studies. The heparin-binding core motif PRRAR is underlined and presented in italics.

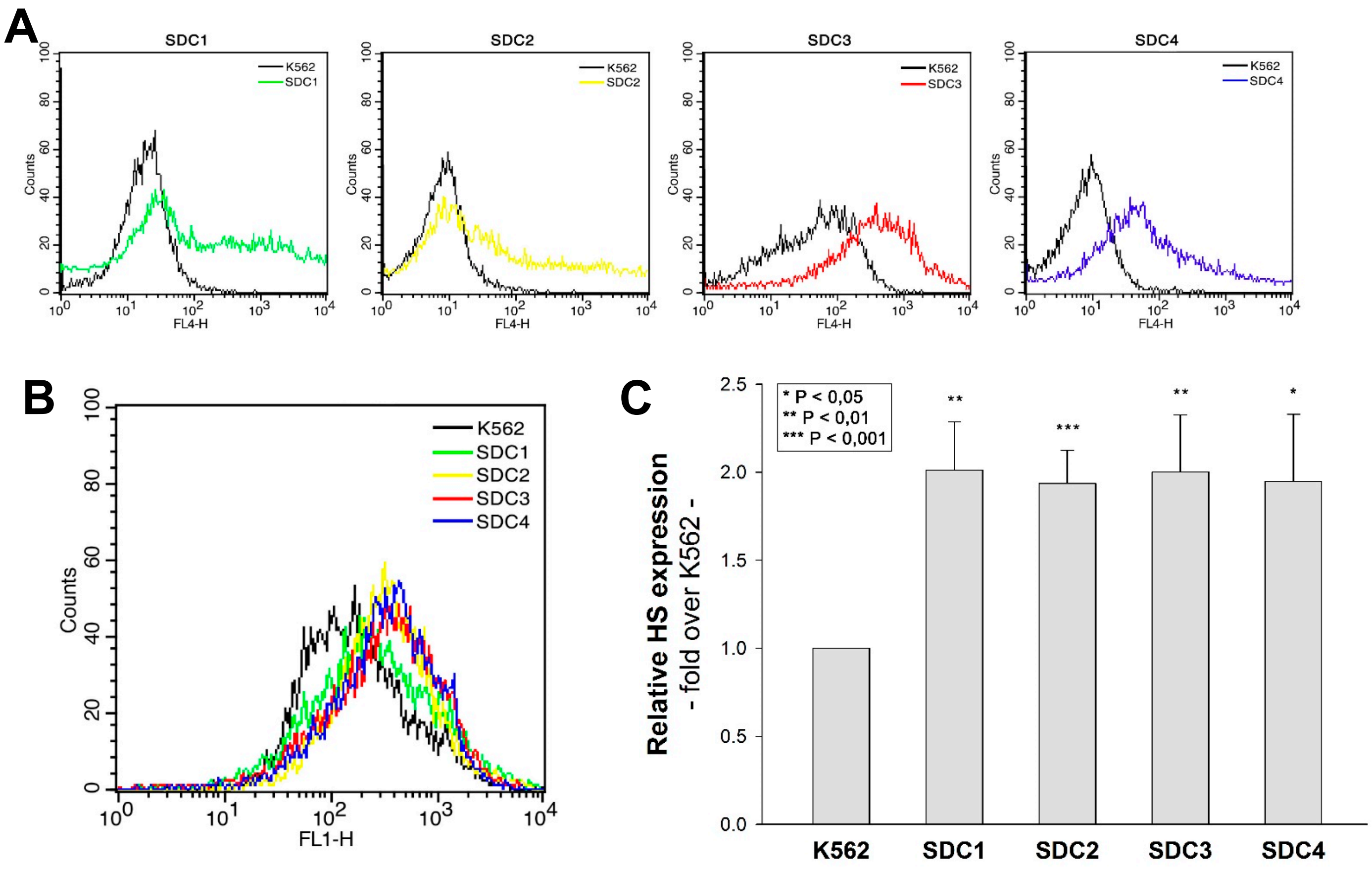

Figure S2. Relative HS expression of SDC transfectants. (**A**) Stable SDC transfectants created in wild-type (WT) K562 cells were selected by measuring SDC expression with flow cytometry (Becton Dickinson FACScan) using APC-labeled anti-SDC antibodies specific for each SDC isoform. (**B**) Flow cytometry histograms showing HS expression of SDC transfectants and WT K562 cells. HS expression of SDC transfectants, along with WT K562 cells, was measured by flow cytometry (Becton Dickinson FACScan) using anti-HS antibody. SDC transfectants with a similar amount of HS expression were selected and applied for further studies. (**C**) Detected HS levels were normalized to WT K562 cells as standards. The bars represent the mean ± SEM of ten independent experiments. Statistical significance vs. WT K562 cells (standards) was assessed with analysis of variance (ANOVA). *p<0.05 vs WT K562 cells; **p<0.01 vs WT K562 cells, ***p<0.001 vs WT K562 cells.

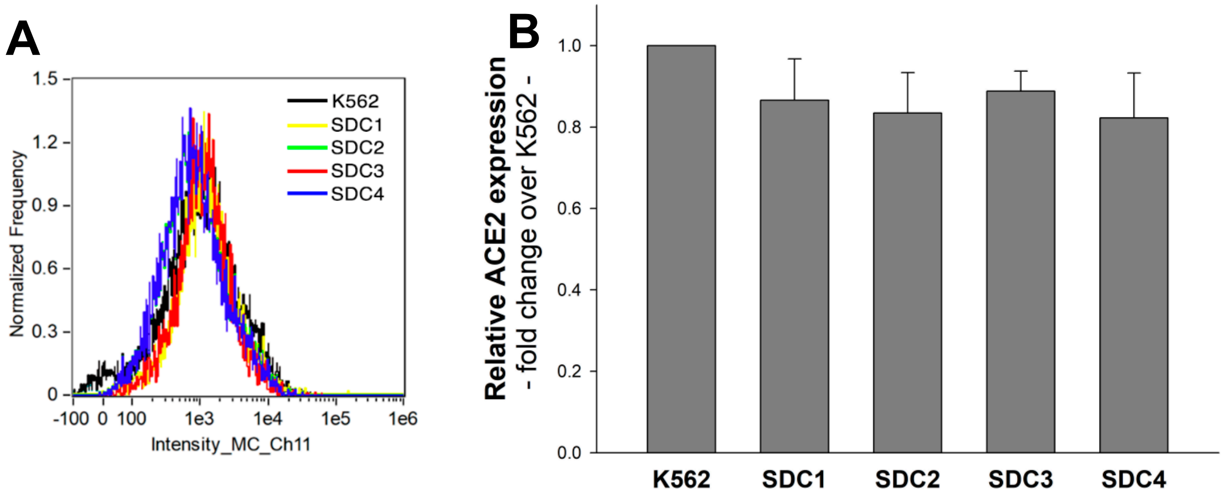

Figure S3. Relative ACE2 expression of K562 cells and SDC transfectants. ACE2 expression of WT K562 and SDC transfectants (created in K562 cells) was measured with flow cytometry using AF 647-labeled ACE2 antibody. (**A**) Representative flow cytometry histograms showing the ACE2 expression of SDC transfectants and WT K562 cells. (**B**) Detected ACE2 levels were normalized to WT K562 cells as standards. The bars represent the mean ± SEM of three independent experiments. Statistical significance vs. WT K562 cells as standards was assessed with analysis of variance (ANOVA). Compared to WT K562 cells, no statistically significant differences were detected in ACE2 expression of SDC transfectants.

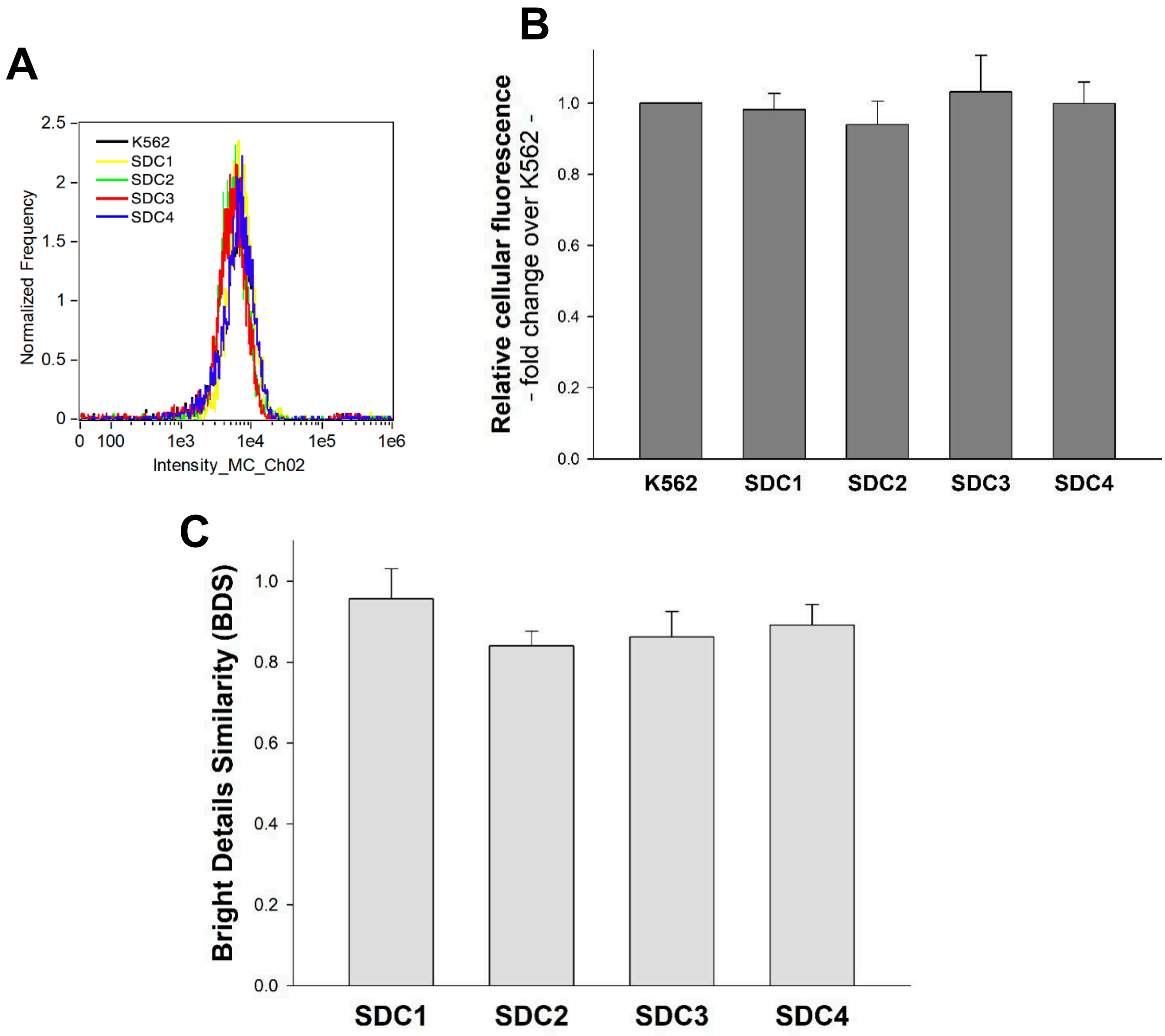

Figure S4. Cellular fluorescence of WT K562 cells and SDC transfectants after AF 488-labeled secondary antibody treatment. WT K562 cells and SDC transfectants were rinsed, fixed, permeabilized, blocked with the appropriate goat serum for 1h at room temperature, followed by AF 488-labeled secondary antibody treatment. After 1 h of incubation, fluorescence of cells was measured with flow cytometry. To measure the potential colocalization of AF 488-labeled secondary antibodies with SDCs, secondary antibody-treated cells were also co-incubated with APC-labeled SDC antibodies. (**A**) Representative flow cytometry histograms showing the fluorescence of WT K562 cells and SDC transfectants incubated with AF 488-labeled goat anti-mouse secondary antibodies. (**B**) Detected fluorescence intensities were normalized to antibody-treated WT K562 cells as standards. The bars represent the mean ± SEM of three independent experiments. Statistical significance vs. standards (WT K562 cells) was assessed with ANOVA. Compared to WT K562 cells, no statistically significant differences were detected. (**C**) Detected BDS scores of AF 488-labeled secondary antibodies and APC-labeled SDCs. The bars represent the mean ± SEM of three independent experiments. Statistical significance between the SDC transfectants was assessed with ANOVA. No statistically significant differences were detected between the SDC transfectants.

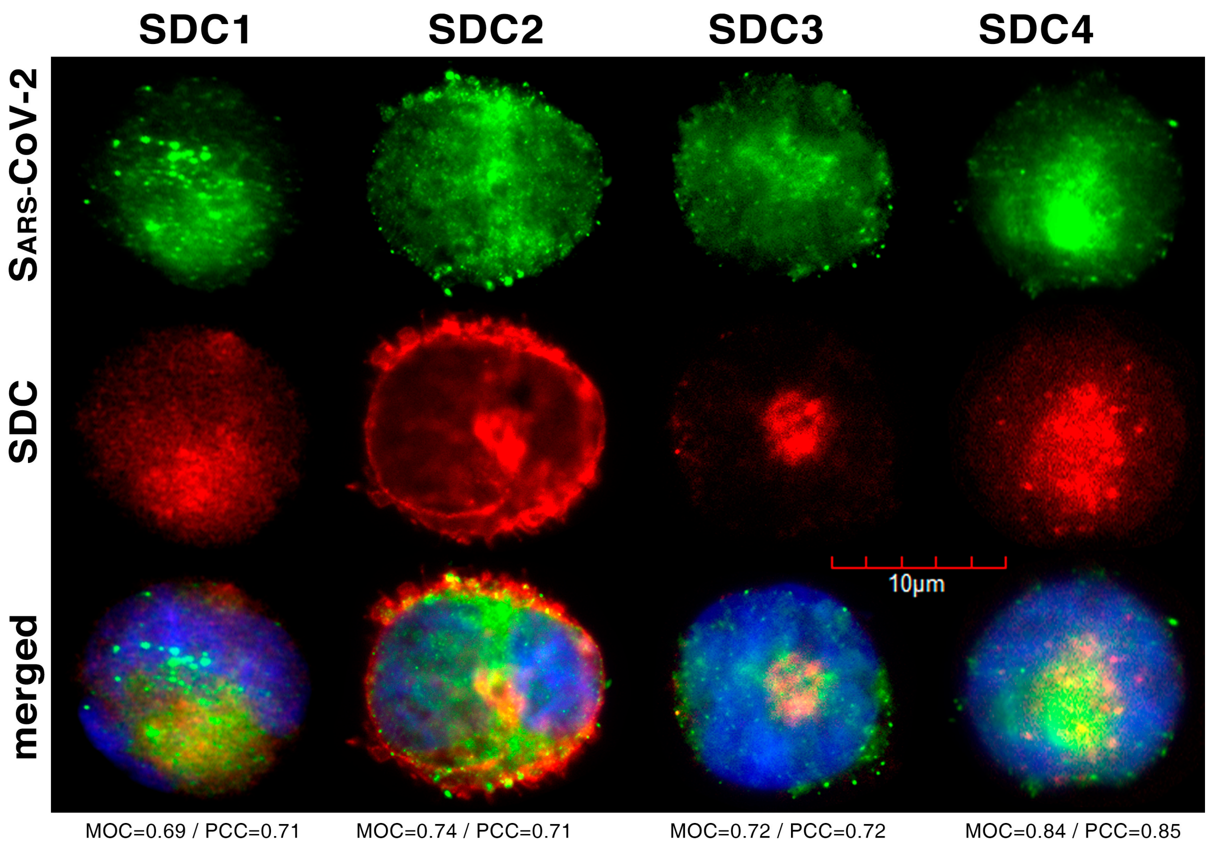

Figure S5. Colocalization of SARS-CoV-2 with SDCs visualized with confocal microscopy. WT K562 cells and SDC transfectants were incubated with heat-inactivated SARS-CoV-2 (at 1 MOI) for 18h at 37°C. After incubation, the cells were washed, trypsinized, fixed, permeabilized and treated with antibodies specific for the spike glycoprotein of SARS-CoV-2 (and AF 488-labeled secondary antibodies) along with APC-labeled SDC antibodies. Colocalization of SARS-CoV-2 with SDCs was then analyzed with confocal microscopy. Representative images of three independent experiments are shown. Scale bar = 10 µm. MOC and PCC values for the overlap and colocalization of SDC with SARS-CoV-2 are indicated below the images.

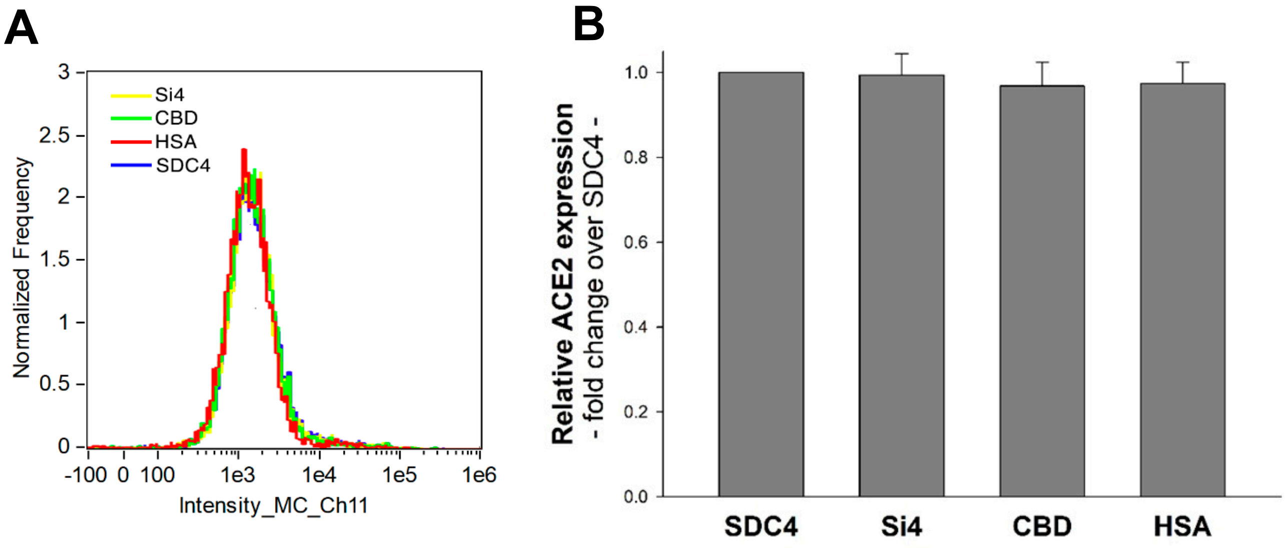

Figure S6. Relative ACE2 expression of SDC4 mutants. ACE2 expression of SDC4 mutants was measured with flow cytometry using AF 647-labeled ACE2 antibody. **(A)** Flow cytometry histograms showing the ACE2 expression of SDC4 mutants. **(B)** Detected ACE2 levels were normalized to SDC4 transfectants as standards. The bars represent the mean ± SEM of three independent experiments. Statistical significance vs. SDC4 transfectants as standards was assessed with ANOVA. Compared to SDC4 transfectants, no significant differences were detected in ACE2 expression.

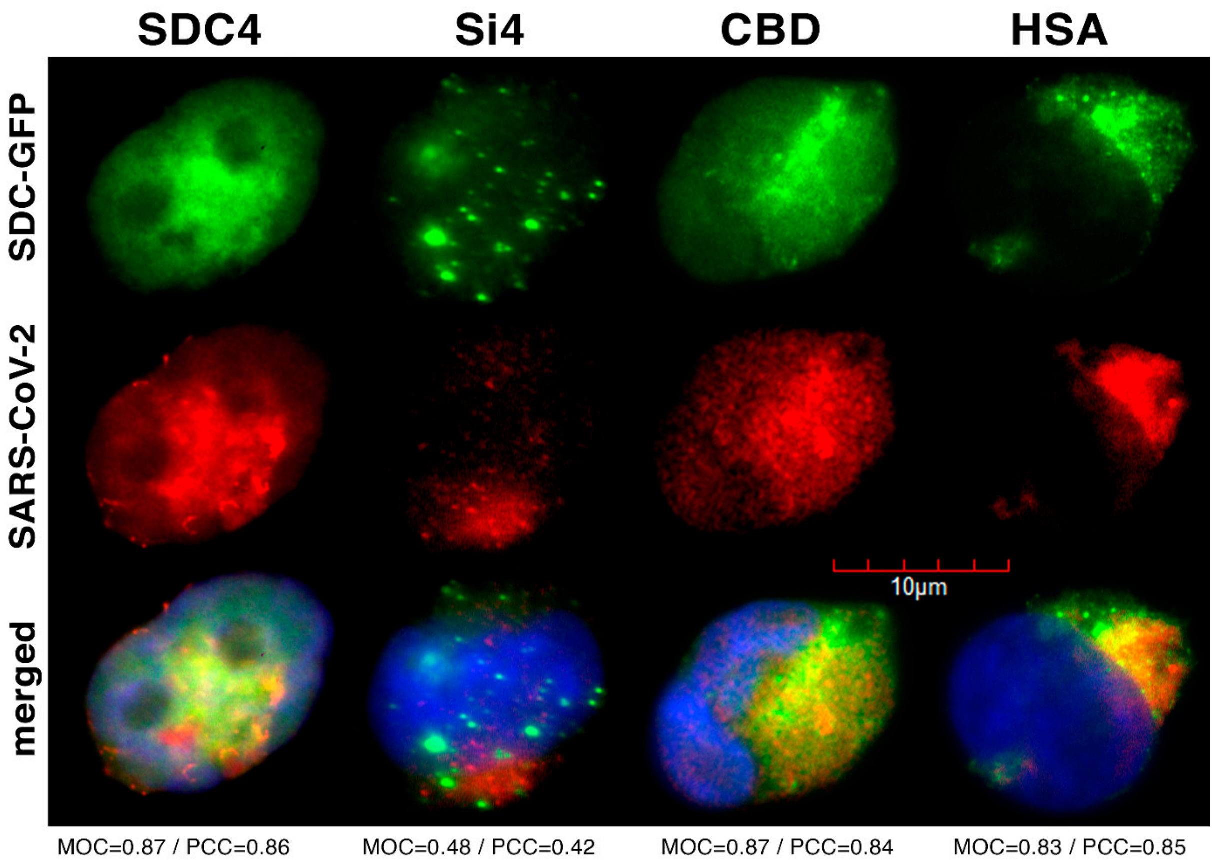

Figure S7. Contribution of the various parts of the SDC4 ectodomain to SARS-CoV-2 uptake. GFP-tagged SDC4 mutants incubated with SARS-CoV-2 (at 1 MOI) for 18 h were fixed, permeabilized and treated with specific and AF 633-labeled antibodies against SARS-CoV-2. Cellular uptake was then analyzed with confocal microscopy. Scale bar = 10 µm. MOC and PCC values for the overlap and colocalization of SARS-CoV-2 with SDC4, Si4, CBD and HSA are indicated below the images.

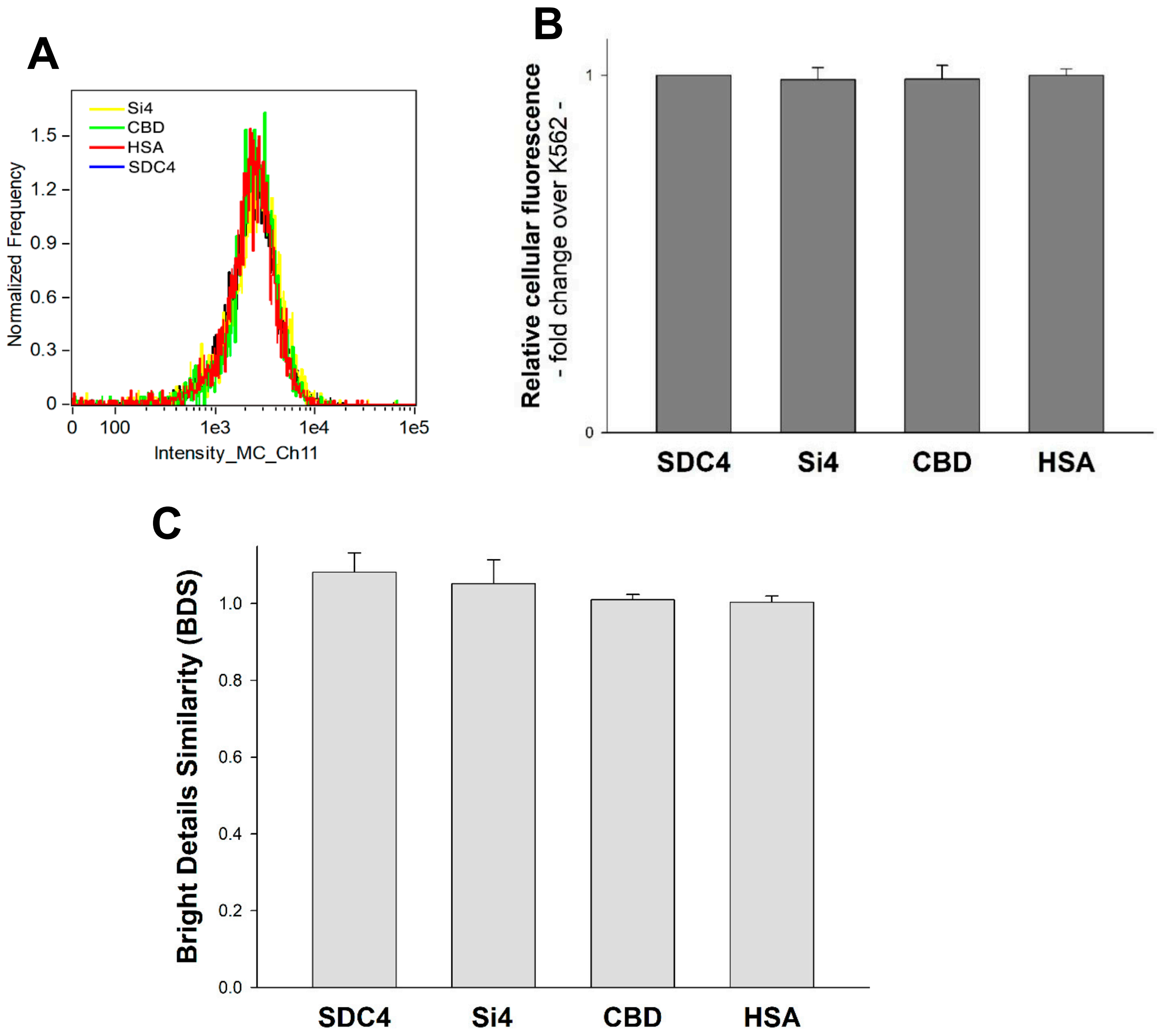

Figure S8. Cellular fluorescence of SDC4 mutants after secondary antibody treatment. SDC4 mutants were washed, rinsed, fixed, permeabilized, and the cells were blocked with serum for 1h at room temperature, followed by AF 633-labeled secondary antibody treatment. After 1 h of incubation, fluorescence of cells was measured with flow cytometry. **(A)** Representative flow cytometry histograms showing the fluorescence of WT K562 cells and SDC transfectants incubated with AF 633-labeled goat anti-mouse secondary antibodies. **(B)** Detected fluorescence intensities were normalized to antibody-treated WT SDC4 transfectants as standards. The bars represent the mean ± SEM of three independent experiments. Statistical significance vs. standards (WT SDC4 transfectants) was assessed with ANOVA. Compared to standards, no statistically significant differences were detected. **(C)** Detected BDS scores of AF 633-labeled secondary antibodies and GFP-tagged SDC4 mutants. The bars represent the mean ± SEM of three independent experiments. Statistical significance between the SDC transfectants was assessed with ANOVA. No statistically significant differences were detected between the SDC4 mutants.

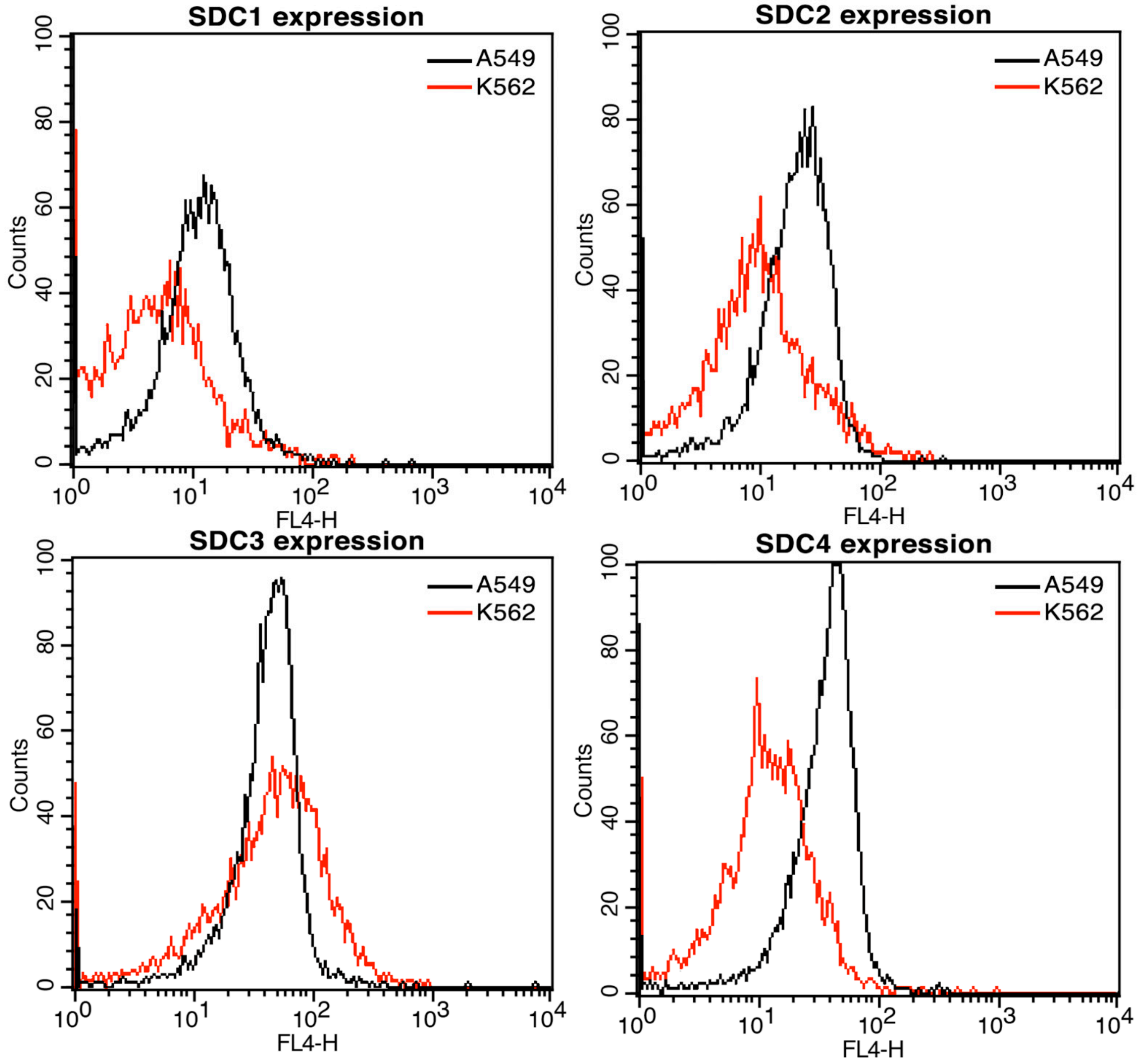

Figure S9. Representative flow cytometry histograms showing the expression levels of SDC isoforms in WT A549 cells. SDC expression of WT A549 and K562 cells was measured with flow cytometry using APC-labeled SDC antibodies as described in Materials and methods.

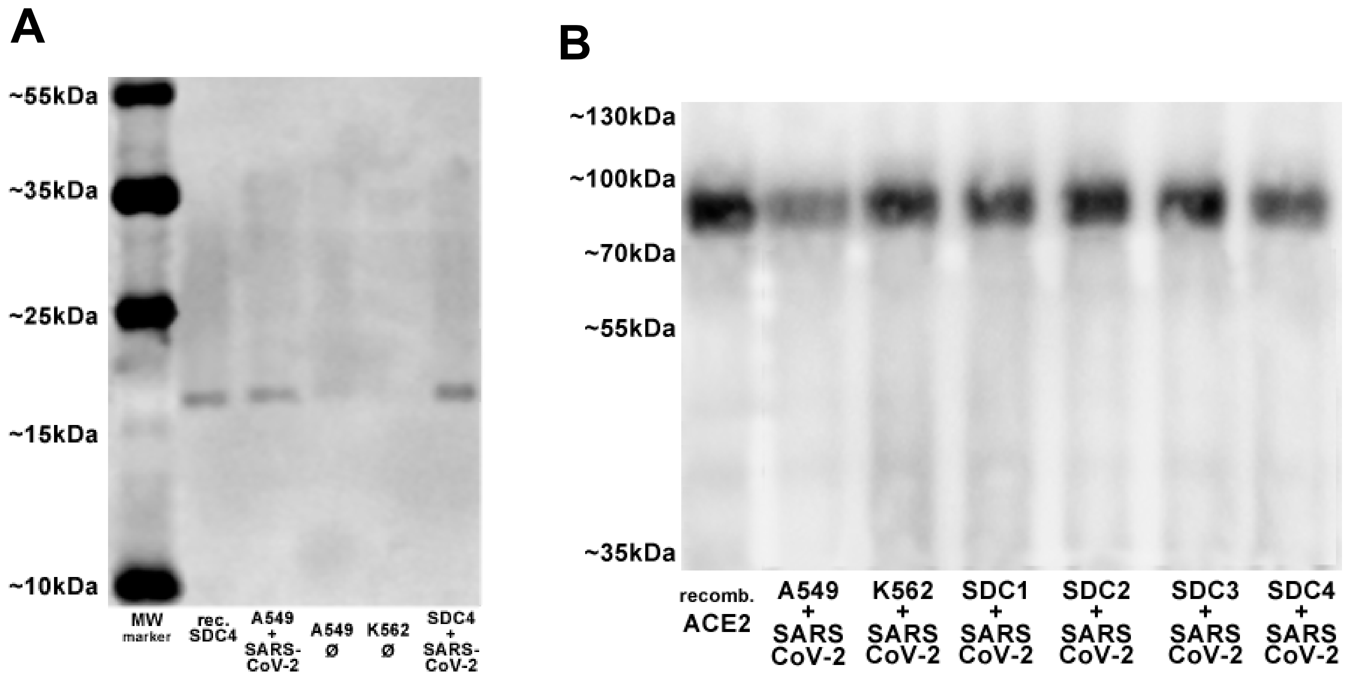

Figure S10. SDC4 and ACE2 immunoprecipitated with SARS-CoV-2. **(A-B)** Representative Western blot analyses showing SDC4 **(A)** or ACE2 **(B)** immunoprecipitated with SARS-CoV-2 in WT A549 cells and SDC4 transfectants. SDC4 **(A)** or ACE2 **(B)** were detected with UVITEC Alliance Q9 Advanced imaging platform. **(A)** Lane 1: 0.5 ug of recombinant SDC4; Lanes 2-3: immunoprecipitates of WT A549 cells treated with or without SARS-CoV-2, respectively; Lane 4: immunoprecipitate of WT K562 cells untreated with SARS-CoV-2. Lane 4: immunoprecipitate of SDC4 transfectants (created in K562 cells) treated with SARS-CoV-2. **(B)** Lane 1: 0.5 ug of recombinant ACE2; Lanes 2-3: immunoprecipitates of SARS-CoV-2-treated WT A549 and K562 cells, respectively; Lane 4-7: immunoprecipitate of SDC transfectants (created in K562 cells) treated with SARS-CoV-2. Standard protein size markers are indicated on the left.

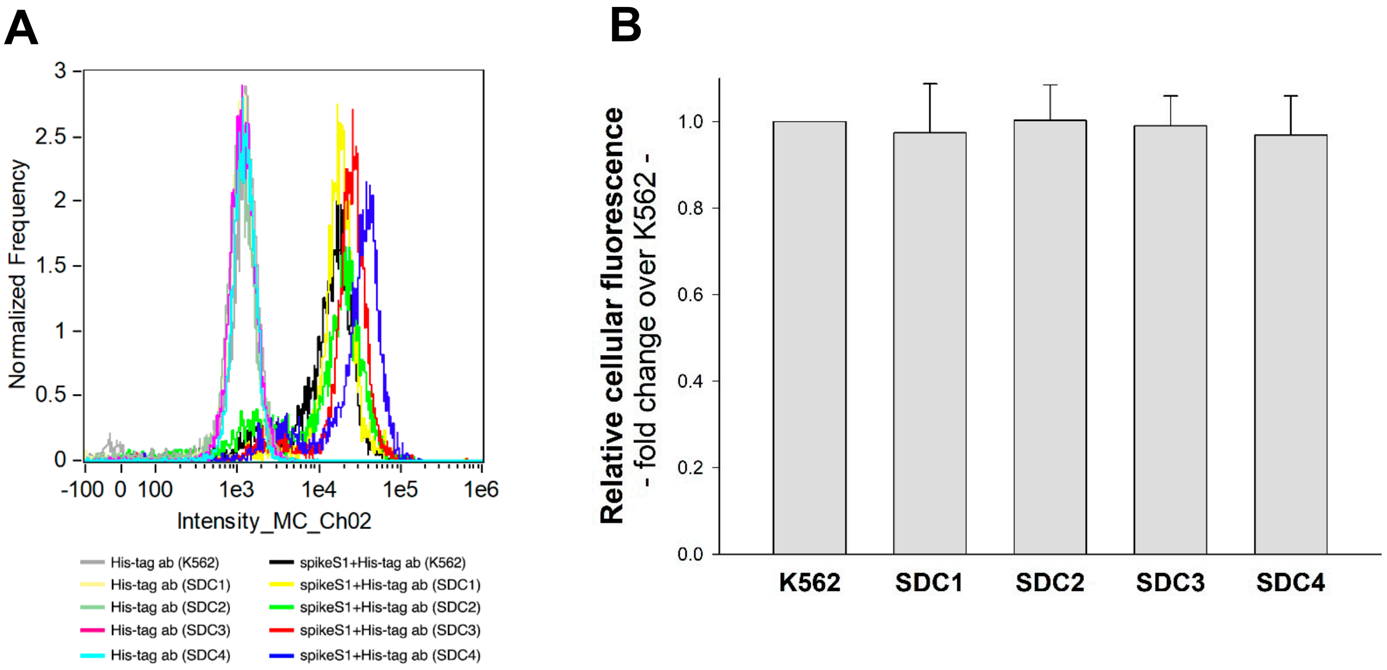

Figure S11. Control studies with anti-His tag antibody-treated WT K562 cells and SDC transfectants. The cells were trypsinized, fixed, permeabilized and treated with the FITC-labeled anti-His-tag antibodies. Cellular fluorescence was then measured with flow cytometry. **(A)** Flow cytometry histograms showing intracellular fluorescence of anti-His tag antibody-treated cells preincubated without spikeS1. For comparison, the histogram also shows the fluorescence of cells of anti-His tag antibody-treated cells preincubated with spikeS1. **(B)** Detected fluorescence intensities of anti-His tag antibody-treated cells preincubated without spikeS1 were normalized to antibody-treated WT K562 cells as standards. The bars represent the mean ± SEM of three independent experiments. Statistical significance vs. standards was assessed with ANOVA. Compared to WT K562 cells, SDC transfectants did not exhibit statistically significant differences in cellular fluorescence after fluorescent anti-His tag antibody treatment.

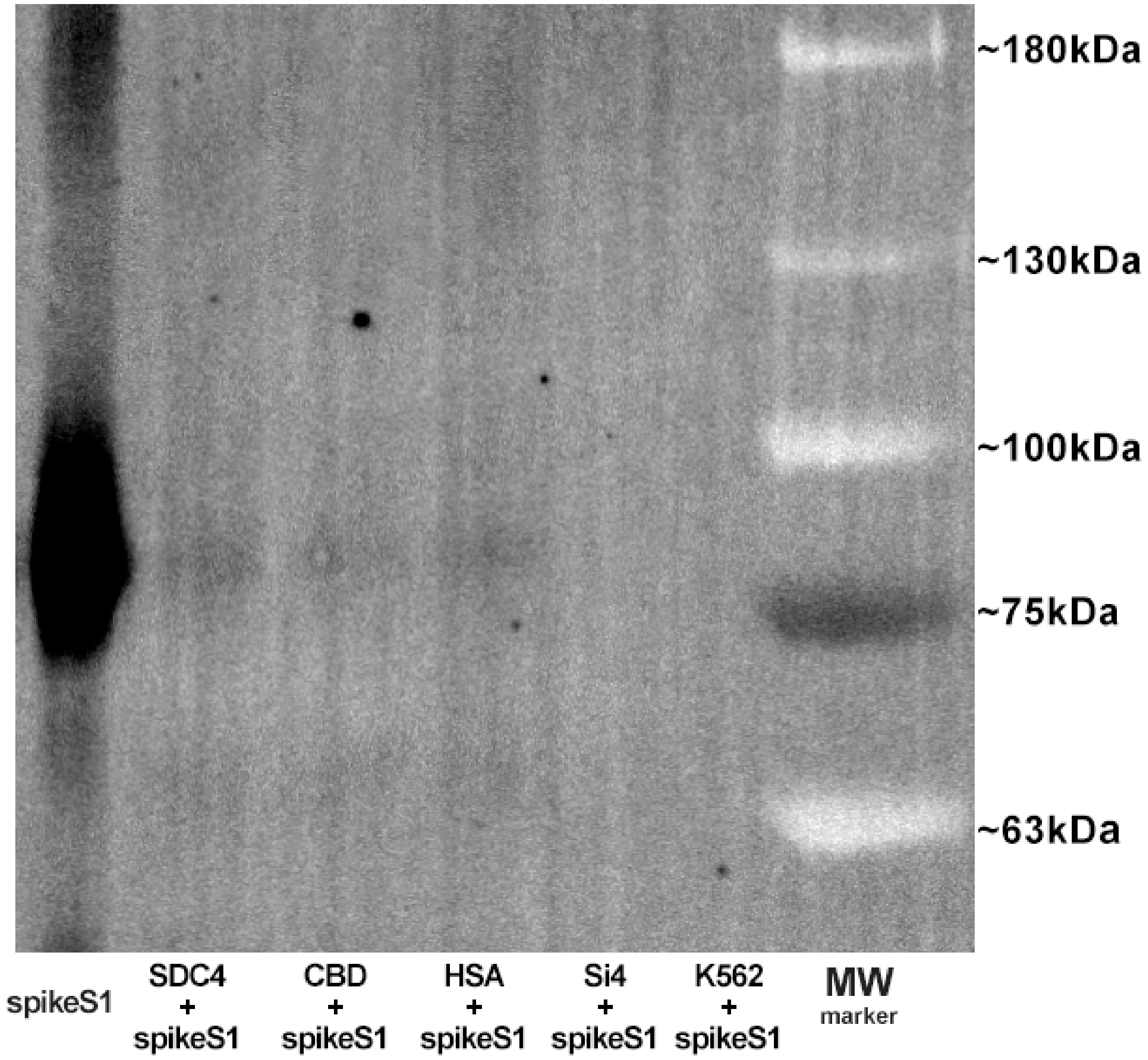

Figure S12. SpikeS1 immunoprecipitated with SDC4 and its mutants. Representative Western blot analysis showing spikeS1 immunoprecipitated with SDC4. SpikeS1 was detected with UVITEC Alliance Q9 Advanced imaging platform. Lane 1: 0.5 ug of spikeS1; Lanes 2-5: immunoprecipitates of SDC4 transfectants, CBD, HSA, Si4 mutants and WT K562 cells treated with spikeS1, respectively. Standard protein size markers are indicated on the right.

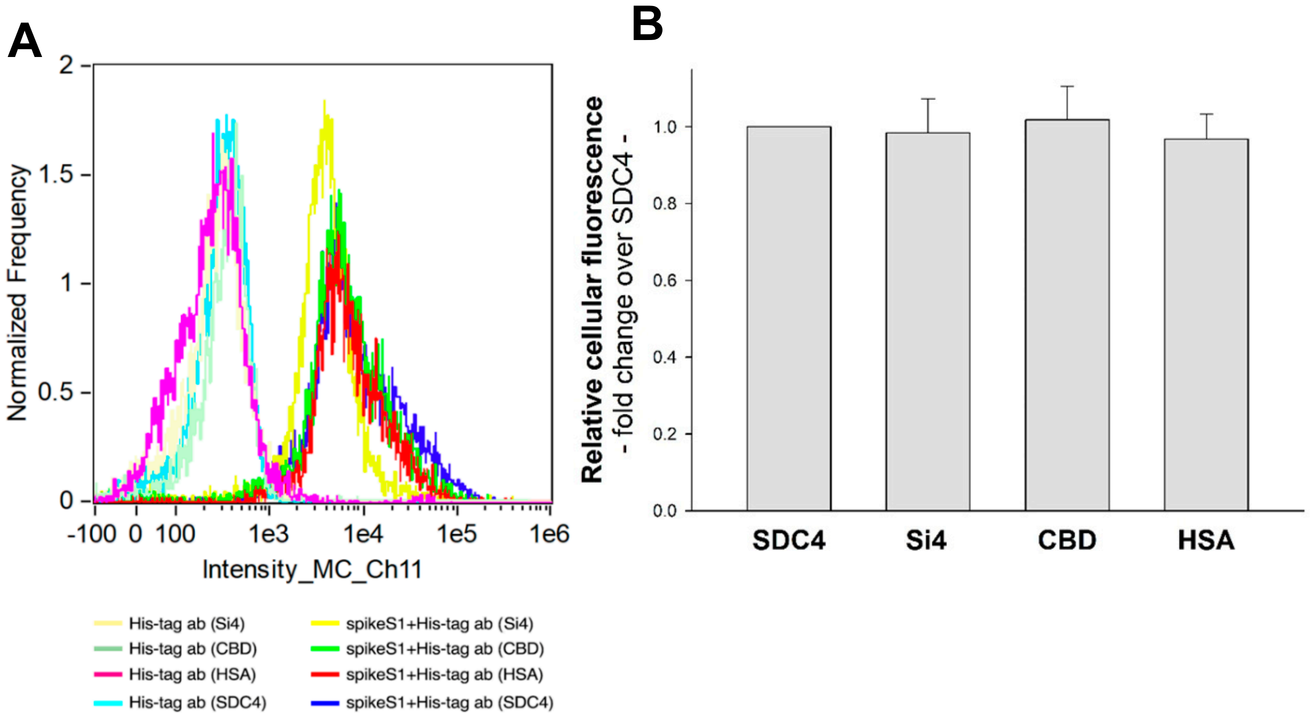

Figure S13. Control studies with anti-His tag antibody-treated SDC4 transfectants and mutants. The cells were trypsinized, fixed, permeabilized and treated with the AF 647-labeled anti-His-tag antibodies. Cellular fluorescence was then measured with flow cytometry. **(A)** Flow cytometry histograms showing intracellular fluorescence of anti-His tag antibody-treated SDC4 transfectants and mutants preincubated with or without spikeS1. **(B)** Detected fluorescence intensities of anti-His tag antibody-treated SDC4 transfectants and mutants preincubated without spikeS1 were normalized to antibody-treated SDC4 transfectants as standards. The bars represent the mean ± SEM of three independent experiments. Statistical significance vs. standards was assessed with ANOVA. Compared to WT SDC4 transfectants, SDC4 mutants did not exhibit statistically significant differences in cellular fluorescence after fluorescent anti-His tag antibody treatment.

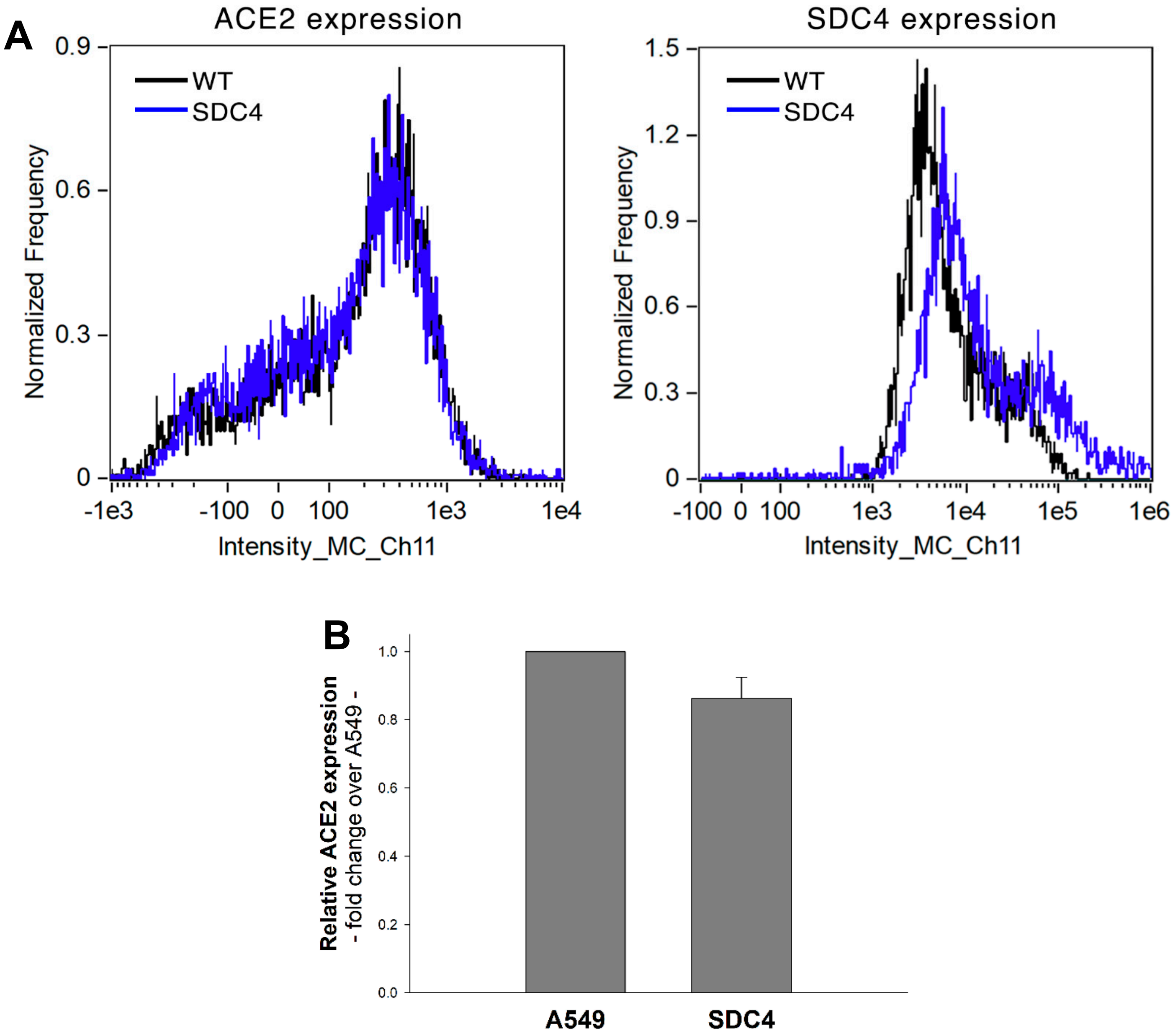

Figure S14. Relative ACE2 expression of A549 cells and SDC4 transfectants. ACE2 expression of WT A549 cells and SDC4 transfectants was measured with flow cytometry using AF 647-labeled ACE2 antibody. **(A)** Representative flow cytometry histograms showing the ACE expression of WT A549 cells and SDC transfectants. **(B)** Detected ACE2 levels were normalized to WT A549 cells as standards. The bars represent the mean ± SEM of three independent experiments. Statistical significance vs. A549 cells as standards was assessed with ANOVA. Compared to WT A549 cells, no statistically significant differences were detected in ACE2 expression.

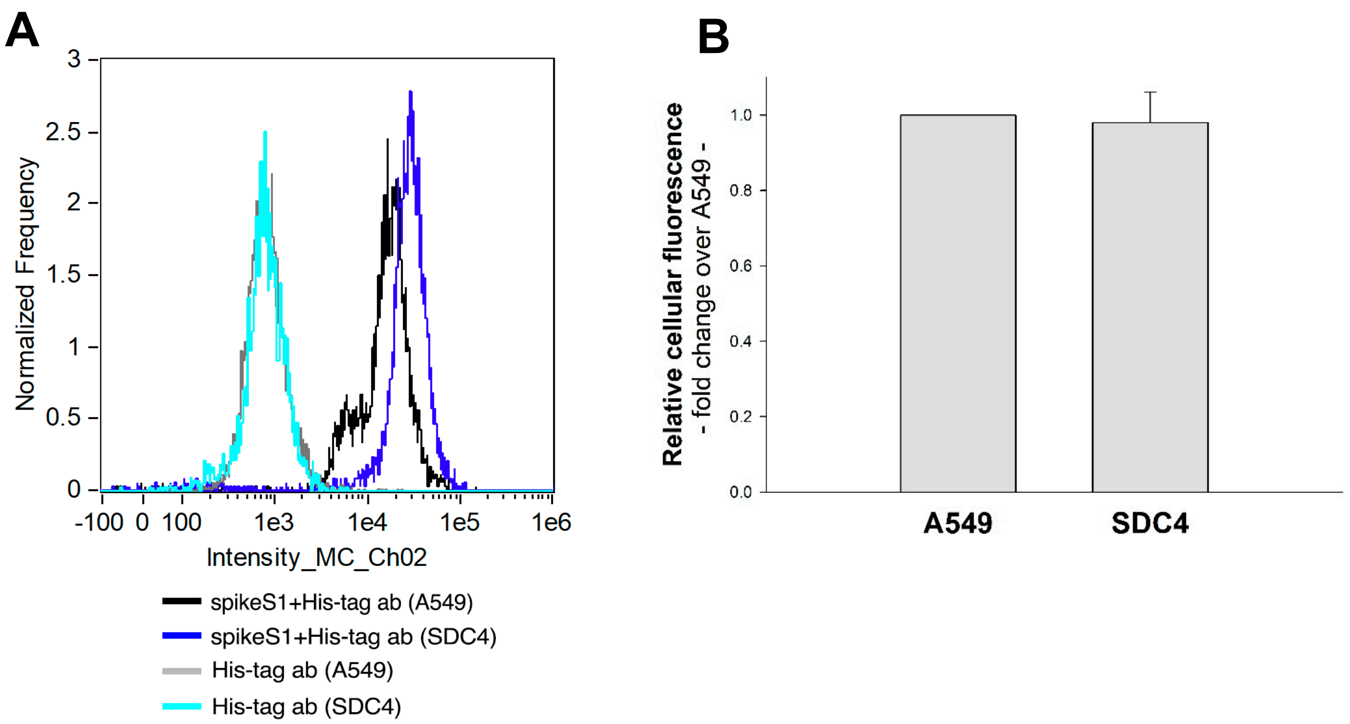

Figure S15. Control studies with anti-His tag antibody-treated WT A549 cells and SDC4 transfectants (created in A549 cells). The cells were trypsinized, fixed, permeabilized and treated with the FITC-labeled anti-His-tag antibodies. Cellular fluorescence was then measured with flow cytometry. **(A)** Flow cytometry histograms showing intracellular fluorescence of anti-His tag antibody-treated WT A549 cells and SDC4 transfectants preincubated with or without spikeS1. **(B)** Detected fluorescence intensities of anti-His tag antibody-treated WT A549 cells and SDC4 transfectants preincubated without spikeS1 were normalized to antibody-treated WT A549 cells as standards. The bars represent the mean ± SEM of three independent experiments. Statistical significance vs. standards was assessed with ANOVA. Compared to WT A549 cells, SDC4 transfectants did not exhibit statistically significant differences in cellular fluorescence after fluorescent anti-His tag antibody treatment.

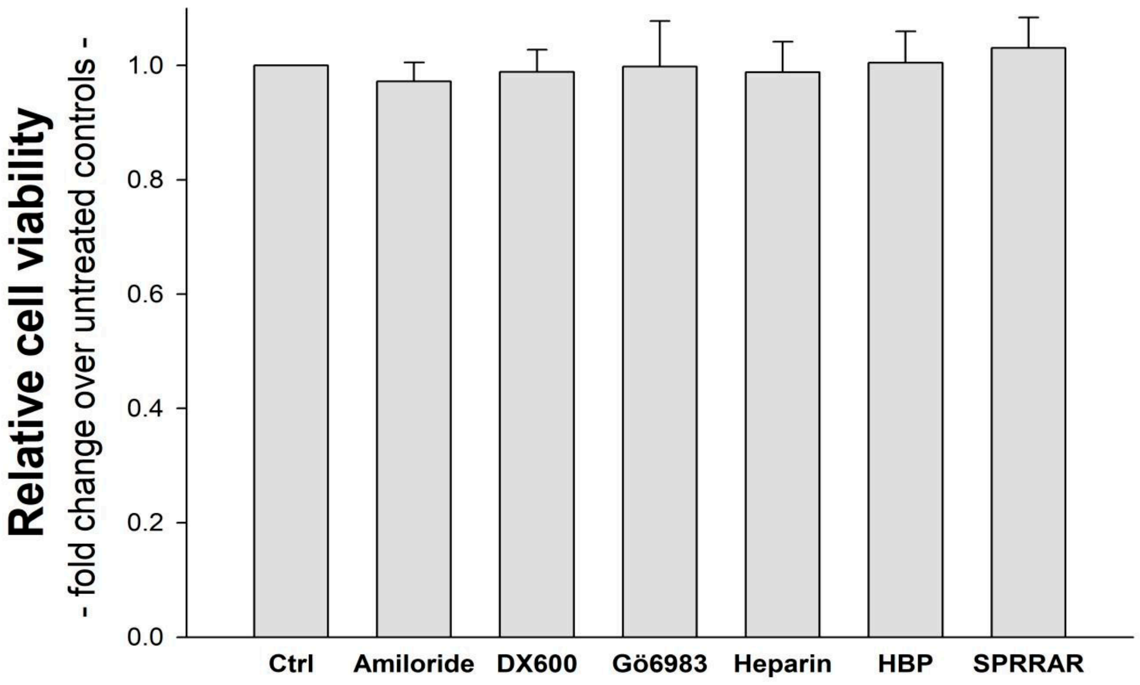

Figure S16. Cellular viability measurements in the presence of the applied inhibitors. WT A549 cells were incubated with or without either of the following inhibitors for 18 h: amiloride (100 µM), DX600 (10 µM), Gö 6983 (10 µM), heparin (200 µg/ml), HBP (100 µM) and SPRRAR (100 µM). Cellular viability was then measured with EZ4U assay and detected measures were then normalized to untreated WT A549 cells as controls (Ctrl). The bars represent the mean ± SEM of three independent experiments. Statistical significance vs. controls was assessed with ANOVA. Compared to controls, no statistically significant differences were detected in the viability of inhibitor-treated cells.

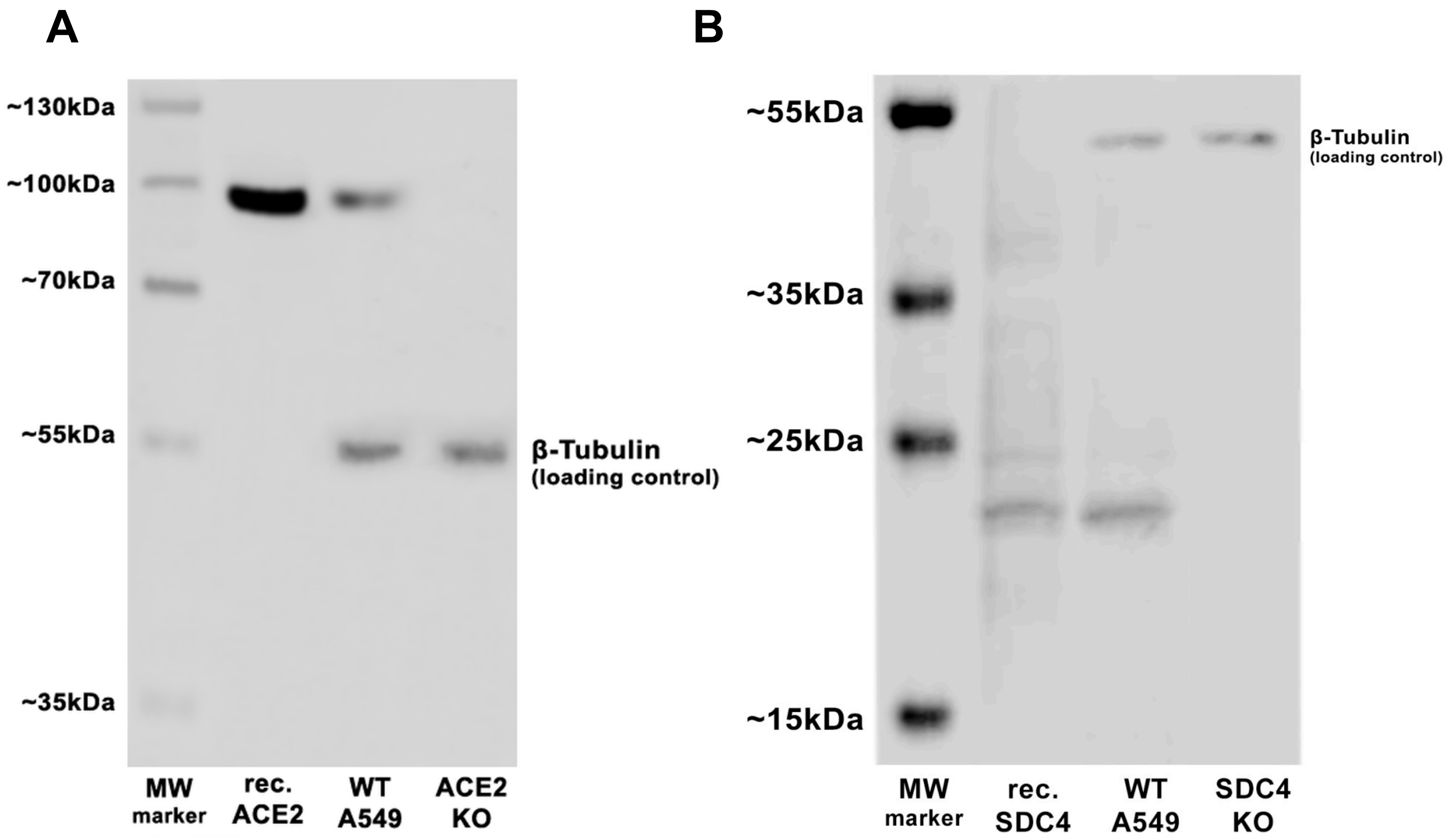

Figure S17. Western blot validation of ACE2 **(A)** and SDC4 **(B)** knockdown in A549 cells. SDC4 and ACE2 knockdown in A549 cells was performed using a lentiviral vector system specific to human ACE2 and SDC4 shRNA. Stable KO cells were selected in 2 mg G418 and sorted using imaging flow cytometry (Amnis FlowSight) with APC-conjugated anti-SDC4 and Alexa Fluor 647-labeled anti-ACE2 antibodies. Cellular expression of ACE2 and SDC4 following knockdown was also determined with Western blotting. WT A549 and SDC4 and ACE2 shRNA-treated cells were grown in 24-well plates for 24 h, then the medium was removed and the cells were washed (with PBS) and lysed in RIPA buffer. Protein concentrations were measured with spectrophotometer (Metertech UV/VIS). Equal amounts of protein from cell lysates were then subjected to SDS-PAGE on 7,5%–12,5% gradient gels and electroblotted onto PVDF membranes using the Mini Wide Vertical Electrophoresis gel system (Cleaver Scientific). The membranes were blocked in TBST with 5% dry milk, washed, incubated with anti-ACE2 and anti-SDC4 antibodies diluted in TBST with 1% dry milk for 2 h, and then incubated with HRP-conjugated secondary antibodies. Chemiluminescence detection reagent was used for protein visualization and the signal was detected with UVITEC Alliance Q9 Advanced Imager. β-Tubulin was used as a loading control.
